# Pre-release environmental acclimation enhances wild adaptability of endangered Kaluga sturgeon (*Huso dauricus*): insights from digestive, immune, and gut-microbiome perspectives

**DOI:** 10.3389/fmicb.2025.1720688

**Published:** 2025-12-04

**Authors:** Cunhua Zhai, Wentao Sun, Yutao Li, Haoxiang Han, Ying Zhang, Bo Ma

**Affiliations:** 1 Heilongjiang River Fishery Research Institute, Chinese Academy of Fishery Sciences, Harbin, China; 2 Research Station for Wild Scientific Observation on Fishery Resources and Ecological Environment Protection, Jiamusi, Ministry of Agriculture and Rural Affairs, Harbin, China; 3 College of Fisheries and Life Science, Shanghai Ocean University, Shanghai, China; 4 Key Laboratory of Cold Water Fish Germplasm Resources and Multiplication and Cultivation of Heilongjiang Province, Heilongjiang River Fishery Research Institute, Chinese Academy of Fishery Sciences, Harbin, China

**Keywords:** *Huso dauricus*, environmental acclimation, digestion, gut microbiota, immunity

## Abstract

**Introduction:**

Pre-release environmental acclimation is an effective strategy for improving post-stocking survival and restoring wild genetic resources in hatchery-reared juveniles. However, environmental acclimation protocols for the endangered Kaluga sturgeon (*Huso dauricus*) are currently non-existent.

**Methods:**

Here, cultured *H. dauricus* were transferred to a tributary of the Songhua River in autumn and exposed to an *in-situ* environmental acclimation protocol for 30 days. Subsequently, a hatchery control (HK) and seven environmental acclimation groups—HC1 (day 2), HC2 (day 5), HC3 (day 10), HC4 (day 15), HC5 (day 20), HC6 (day 25) and HC7 (day 30)—were monitored for feeding rate, digestive and immune enzyme activities, immune-gene expression, and gut microbiota change.

**Results:**

During the initial phase of wild conditioning, feeding rate remained negligible until HC2 group, then increased to 66%, 88.89% and 100% in groups HC4, HC5 and HC6, respectively. Meanwhile, digestive enzyme activities stabilized between groups HC4 and HC5, and immune enzyme activities in the wild-conditioned sturgeon were markedly higher than those of the control group. In addition, compared with the control group, the pro-inflammatory cytokine *Interleukin-6* (*IL-6*) was significantly up-regulated, whereas the anti-inflammatory genes *Interleukin-10* (*IL-10*) and *Transforming growth factor-beta* (*TGF*-β) were significantly down-regulated in HC4 group (*P* < 0.05). At the phylum level, the dominant microbiota shifted from Pseudomonadota to Bacillota by day 20 (HC5 group) and thereafter remained stable.

**Discussion:**

This study provides a theoretical framework for characterizing the physiological and biochemical responses of *H. dauricus* during environmental acclimation and provides a scientific basis for conserving its wild genetic resources.

## 1 Introduction

Kaluga sturgeon (*Huso dauricus*) is a relict species that first appeared during the Cretaceous period and has persisted for approximately 130 million years, earning it the epithet “living fossil” (Wei et al., 1997; Zhuang et al., 2022; Liu et al., 2010). Owing to its ancient lineage and exceptional biological traits, the species is of outstanding economic importance and serves as a key model for evolutionary and conservation research (Song et al., 2023; Koshelev et al., 2022). In China, *H. dauricus* is distributed primarily in the large rivers and lakes of Heilongjiang Province, with additional records from the Songhua River (flowing through Jilin and Heilongjiang provinces) and the Ussuri River, which flows north into Russia. Overfishing and escalating anthropogenic disturbances (Su et al., 2021) have driven continuous declines in wild *H. dauricus* populations over the past decade (Cámara-Ruiz et al., 2019; Rochard et al., 1990). Consequently, *H. dauricus* was listed in Appendix II of the Convention on International Trade in Endangered Species in 1998 and is currently classified as Critically Endangered, thereby mandating urgent conservation measures (Ye and Valbo-Jørgensen, 2012; Wei and Ruban, 2010).

To counteract the endangered status of *H. dauricus*, current conservation measures rely on hatchery-based restocking programmes and fishing moratoria both within China and internationally (Gessner et al., 2011; Li J. et al., 2021). Nevertheless, direct stocking exhibits low efficacy, with only a small fraction of released individuals surviving (Beamish et al., 1992; Nickleson, 1986; Coleman et al., 1998; Pearcy, 1992). According to available statistics, empirical survival rates are < 5% for salmonids, 1%−3% for Pacific salmon and < 1% for cod (McNeil, 1991; Salvanes, 2001). *H. dauricus* faces similar challenges, with empirical evidence showing that post-release survival of stocked juveniles rarely exceeds 3% (Li J. et al., 2021; Chebanov et al., 2011. Predation and starvation are the primary causes of mortality in stocked fish (Cowx, 2025; Humphries et al., 2020). Numerous studies have demonstrated that hatchery-reared juveniles exhibit poor environmental adaptation and consequently low feeding rates, ultimately compromising stocking success (Tomiyama et al., 2011; Le Vay et al., 2007; Steven, 2010). Evidence indicates that pre-release environmental acclimation enhances post-stocking survival and markedly improves the efficacy of restocking programmes for endangered fishes (Brown and Day, 2002). Prolonged pre-release conditioning progressively increases both growth (Hutchison et al., 2023) and recapture rates (Chester et al., 2025), demonstrating that environmental acclimation prior to stocking substantially enhances adaptive capacity and post-release survival, and thus constitutes a key strategy for improving restocking success.

The gut microbiota is a complex and dynamic community that plays a pivotal role in host nutrition and health (Banerjee and Ray, 2017; Feng et al., 2018). These microorganisms modulate immunity, energy storage and nutrient absorption (Bacanu and Oprea, 2013; Wen et al., 2021; De Vos et al., 2022), and their assemblage in fish is a multifaceted process shaped by host genetics as well as environmental conditions (Bolnick et al., 2014; Dehler et al., 2017; Sullam et al., 2012; Ringø et al., 2016; Pinos et al., 2025). Studies have demonstrated that the compositional and functional profiles of fish gut microbiota differ markedly between aquaculture and natural environments, as reported for hatchery-reared vs. wild Kaluga sturgeon (*Huso dauricus*), common carp (*Cyprinus carpio*), Atlantic cod (*Gadus morhua* L.), and Pike perch (*Sander Lucioperca*) (Lv et al., 2018; Ruzauskas et al., 2021; Dhanasiri et al., 2011; Wang et al., 2024). Such microbiota disparities may compromise the survival of artificially propagated fish after their release into the wild. Characterizing gut microbial assemblages is now recognized as a novel tool for the conservation of wild populations (Chong et al., 2019). Investigating the microbiota of cultured and environmentally acclimated *H. dauricus* can therefore elucidate the mechanisms underpinning adaptation to natural conditions and inform strategies to enhance post-release survival in restocking programmes.

**Figure 1:**
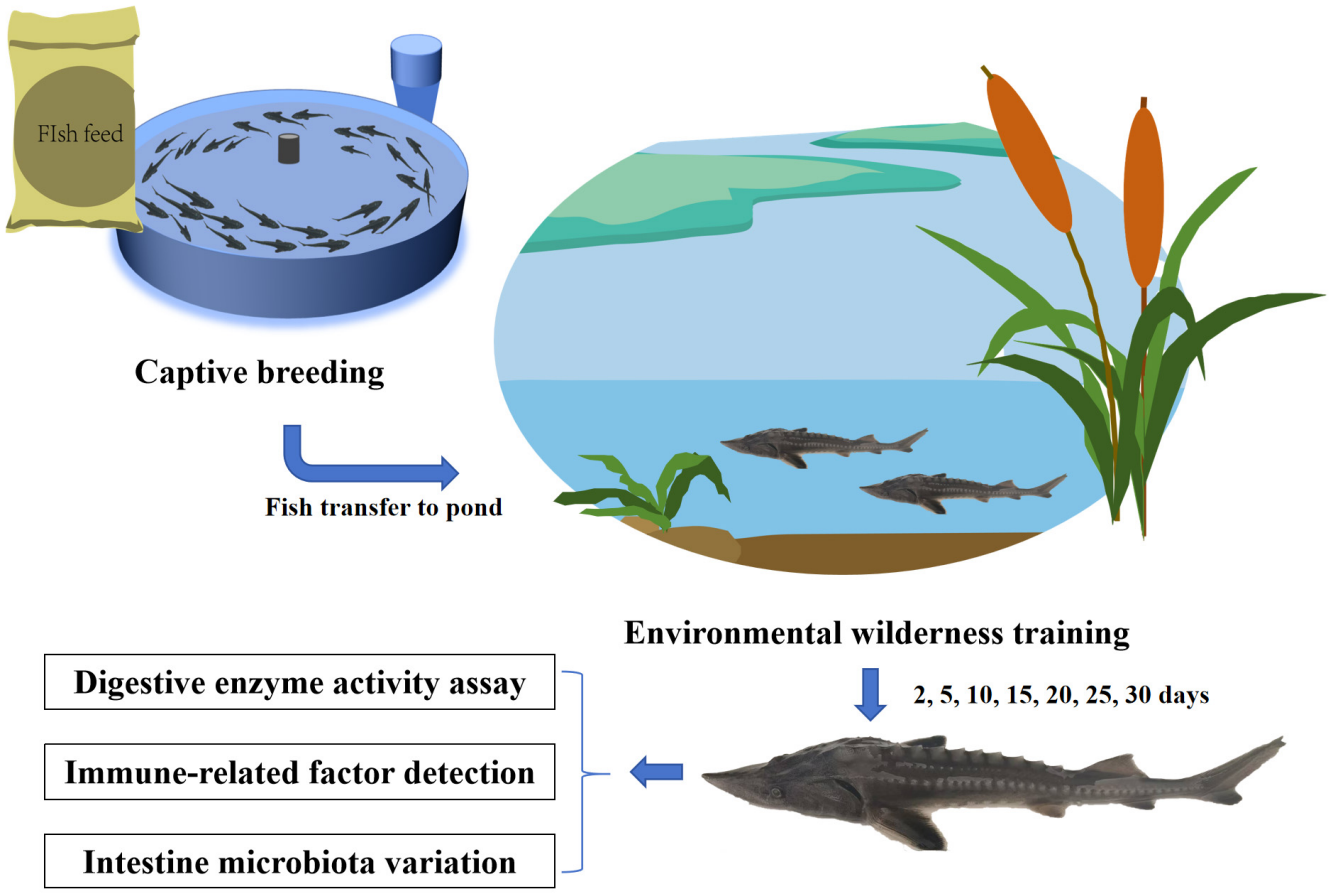
Experimental specimens and schematic diagram for the study design.

Here, we integrated diet analysis, enzyme assays, immune-gene expression profiling and high-throughput sequencing of gut microbiota to (i) track feeding performance, (ii) compare digestive and immune phenotypes, and (iii) characterize microbial community dynamics of *H. dauricus* throughout the 30 day environmental acclimation training (Figure 1). This study aims to identify phenotypic and microbiota difference between hatchery-reared and environmentally acclimated *H. dauricus*, thereby providing a mechanistic framework for the transition from culture to wild-type traits. The findings will provide theoretical underpinnings for strategies to enhance restocking success of *H. dauricus* and offer novel insights into the conservation and genetic-resource augmentation of wild populations.

## 2 Material and methods

### 2.1 Animals breeding and samples collection

All animal experimental procedures were in accordance with the guidelines of the Laboratory Animal Ethics Committee of the Research Institute of Fisheries of the Heilongjiang River (No. 20240823-001) and guidelines of EU Directive 2010/63/EU for animal experiments were followed during the entire experimental work.

Four-month-old *H. dauricus* juveniles were obtained from the Hulan Experimental Station of Heilongjiang River Fisheries Research Institute, Chinese Academy of Fishery Sciences. The control group (HK group) of *H. dauricus* was maintained at the experimental station and fed a commercial diet, whose detailed nutritional composition is given in Supplementary Table S1. Another part of *H. dauricus* was transferred to a pond located on a tributary of the Songhua River (119°52′-132°31′ E, 44°12′-46°38′ N) for environmental acclimation training. The stocking density was 87.8 kg per cubic meter at the start of the experiment. The water-quality parameters of the pond and the culture station are summarized in Supplementary Table S2. At 2 (HC1 group), 5 (HC2 group), 10 (HC3 group), 15 (HC4 group), 20 (HC5 group), 25 (HC6 group), and 30 (HC7 group) days of environmental acclimation, nine individuals of *H. dauricus* were randomly sampled at each time point, anesthetized using 0.02% Methane Sulfonate-222 (MS-222; Sigma, USA), and immediately measured for total length and body weight (Supplementary Table S3). After surface disinfection with 75% ethanol, the entire intestine was aseptically excised and rinsed with sterile 0.75% saline. The organ was then divided into two portions: one was snap-frozen in liquid nitrogen and stored at −80 °C for quantitative real-time PCR (qRT-PCR) and gut microbiome analysis; the other was obtained to determine digestive and immune enzyme activities. The stomach samples were immediately bagged, labeled, chilled on ice, and transported to the laboratory for subsequent analyses.

### 2.2 Feeding rate assessment

The stomach was aseptically removed from *H. dauricus*, incised along the greater curvature, and all gastric contents were gently transferred into a sterile Petri dish. The gastric contents were rinsed with sterile 0.75 % saline to remove mucus and residual digestive fluid, then briefly placed on sterile filter paper to eliminate excess moisture. Next, contents were identified to the lowest possible taxon and enumerated under a stereomicroscope (Leica S6D), and their wet mass was recorded to the nearest 0.0001 g on an analytical balance. For gastric contents degraded beyond visual identification, total DNA was extracted using the Ezup column kit (Sangon Biotech, Shanghai, China), amplified by PCR using universal primers (LCO1490: 5′-GGTCAACAAATCATAAAGATATTGG-3′; HCO2198: 5′-TAAACTTCAGGGTGACCAAAAAATCA-3′) (Xu et al., 2023), and Sanger-sequenced (Sangon Biotech, Shanghai, China). Detailed cycle parameters were set at 95 °C for 180 s, 40 cycles of 95 °C for 5 s, 60 °C for 15 s, and 72 °C for 30 s. The resulting sequences were queried against the NCBI nucleotide database using BLAST and species were assigned on the basis of ≥ 98% identity to reference sequences. Feeding rate was calculated as: Feeding rate (%) = (Number of stomachs containing food/Total number of stomachs examined) × 100%.

### 2.3 Digestive and immune enzyme activity assays

The activities of amylase (JL-T0701), lipase (JL-T1341), trypsin (JL-T1273), acid phosphatase (ACP, JL-T1094) and lysozyme (LZM, JL-T1062) were determined with commercial kits purchased from Jianglai Bio-Technology Co., Ltd. (Shanghai, China) following the manufacturer's instructions. Firstly, 0.1 g of frozen tissue was homogenized on ice in pre-cooled extraction buffer and centrifuged at 12,000 rpm for 10 min at 4 °C, and the supernatant was used for assays. Subsequently, absorbance was recorded at 540 nm (amylase), 405 nm (lipase, trypsin and ACP) and 530 nm (lysozyme) with a microplate reader. Finally, enzyme activities were normalized to wet tissue mass. Detailed definitions and units for each enzyme activity measured could be referred to Supplementary Appendix 1.

### 2.4 Quantitative real-time PCR analysis

To assess the impact of environmental acclimation on the immune competence of *H. dauricus*, a panel of immune-related genes was subjected to qRT-PCR analysis. Primer pairs were designed with Primer Premier 6.0 and synthesized by Sangon Biotech Co., Ltd. (Shanghai, China; Table 1). The β*-actin* gene was selected as the endogenous reference. First-strand cDNA was synthesized using PrimeScript™ RT Reagent Kit with gDNA Eraser (TaKaRa, Japan). Each 10 μL qRT-PCR reaction contained 5 μL 2 × TB Green Premix Ex Taq II (Tli RNaseH Plus), 0.4 μL each of forward and reverse primers (10 μM), 1 μL template cDNA, 0.2 μL 50 × ROX Reference Dye II (Roche, Switzerland), and 3 μL nuclease-free water. qRT-PCR was performed on an ABI 7,500 Real-Time PCR System (Thermo Fisher Scientific, USA) using the following program: 95 °C for 3 min, followed by 40 cycles of 95 °C for 5 s, 60 °C for 15 s, and 72 °C for 30 s. Relative gene expression levels were calculated by the 2^−Δ*ΔCT*^ method.

**Table 1:** The primer sequence of qRT-PCR.

**Gene**	**Primer sequence**	**Product length (bp)**	**Accession number**
*Il-10*	F:AGAGGAGAAATGGTCGTGCC R:AAGCCCTCCACAAATGAGCA	94	XM_073915878
*TGF-β*	F: GGGAGACACAGAGACGATACAG R: TTTTGCGTGAGGTGTTTGGG	101	XM_051399565
*TNF-α*	F:CACACTGGGCTCTTCTTCGT R:GGACTCAGCATCACCGTAGT	183	NM_001124357.1
*IL-1β*	F:GGTTGGTTTATCAGCACCGC R:GCGCTGAAGAGGAGACTGAA	144	XM_073913694.1
*Il-6*	F:GCCATCCGCTCAGAAAACAG R:CATACTGCTGAACACGGGGA	94	NM_001261449.1
*C3*	F:GGCCATGCTTTGCGATCTG R:TGAGGAGGCGGTTCTGAAG	166	NM_000064.4

### 2.5 16S ribosomal DNA sequencing and gut microbiome analysis

Intestinal contents were collected from three individuals per experimental group, and total DNA was extracted using the E.Z.N.A.^®^ Soil DNA Kit (Omega Bio-tek, Norcross, GA, USA) according to the manufacturer's instructions. DNA purity and integrity were assessed with a NanoDrop 2000 spectrophotometer (Thermo Scientific, USA). The V3-V4 region of the 16S rDNA was amplified using primers 338F (5′-ACTCCTACGGGAGGCAGCAG-3′) and 806R (5′-GGACTACHVGGGTWTCTAAT-3′) on a GeneAmp^®^ 9700 thermal cycler (Applied Biosystems, USA). Purified amplicons were pooled in equimolar amounts and paired-end sequenced on an Illumina Nextseq2000 platform (Illumina, San Diego, USA) according to the standard protocols by Majorbio Bio-Pharm Technology Co. Ltd. (Shanghai, China). Raw FASTQ files were de-multiplexed using an in-house perl script, and then quality-filtered by fastp version 0.19.6 and merged by FLASH version 1.2.7 with the following criteria: (i) the reads were truncated at any site receiving an average quality score of < 20 over a 50 bp sliding window, and the truncated reads shorter than 50 bp were discarded, reads containing ambiguous characters were also discarded; (ii) only overlapping sequences longer than 10 bp were assembled according to their overlapped sequence. The maximum mismatch ratio of overlap region is 0.2. Reads that could not be assembled were discarded; (iii) Samples were distinguished according to the barcode and primers, and the sequence direction was adjusted, exact barcode matching, 2 nucleotide mismatch in primer matching. The operational taxonomic units (OTUs) were then clustered at 97% identity with the UPARSE algorithm implemented in VSEARCH (v2.7.1). Subsequently, OTU sequences were classified into corresponding species categories using Bayesian model, and the microbe taxa composition was analyzed at the phylum, family and genus levels. Venn diagrams were generated to identify unique and shared OTUs between the HK and HC groups. Alpha diversity (Chao1, Shannon, and Simpson indices) was calculated with QIIME (v1.8.0). Beta-diversity was assessed by principal-coordinate analysis (PCoA) based on unweighted UniFrac distances derived from the OTU and taxonomic abundance tables. Functional profiles and COG pathway predictions were inferred using PICRUSt2 (v2.2.0). All bioinformatics analyses were performed on the Majorbio Cloud Platform (https://www.majorbio.com).

### 2.6 Statistics analysis

All results were presented as mean ± standard deviation (SD) and the data was analyzed by the SPSS statistical software (Version 22.0). Results were checked for normal distribution (Shapiro-Wilk test) and variance homogeneity (Levene test). When both assumptions were met, one-way ANOVA followed by Duncan's multiple-range test was employed for inter-group comparisons. If variances were heterogeneous (Levene's test, *P* < 0.05), Welch's ANOVA and the Tamhane T2′s procedure were applied instead. *P* < 0.05 was considered as the significance threshold. The bacterial relative abundances were analyzed using the Kruskal-Wallis test with false discovery rate (FDR) correction for multiple testing. All data was visualized using GraphPad Prism 8.4.3 (686).

## 3 Results

### 3.1 Feeding performance during environmental acclimation

**Figure 2:**
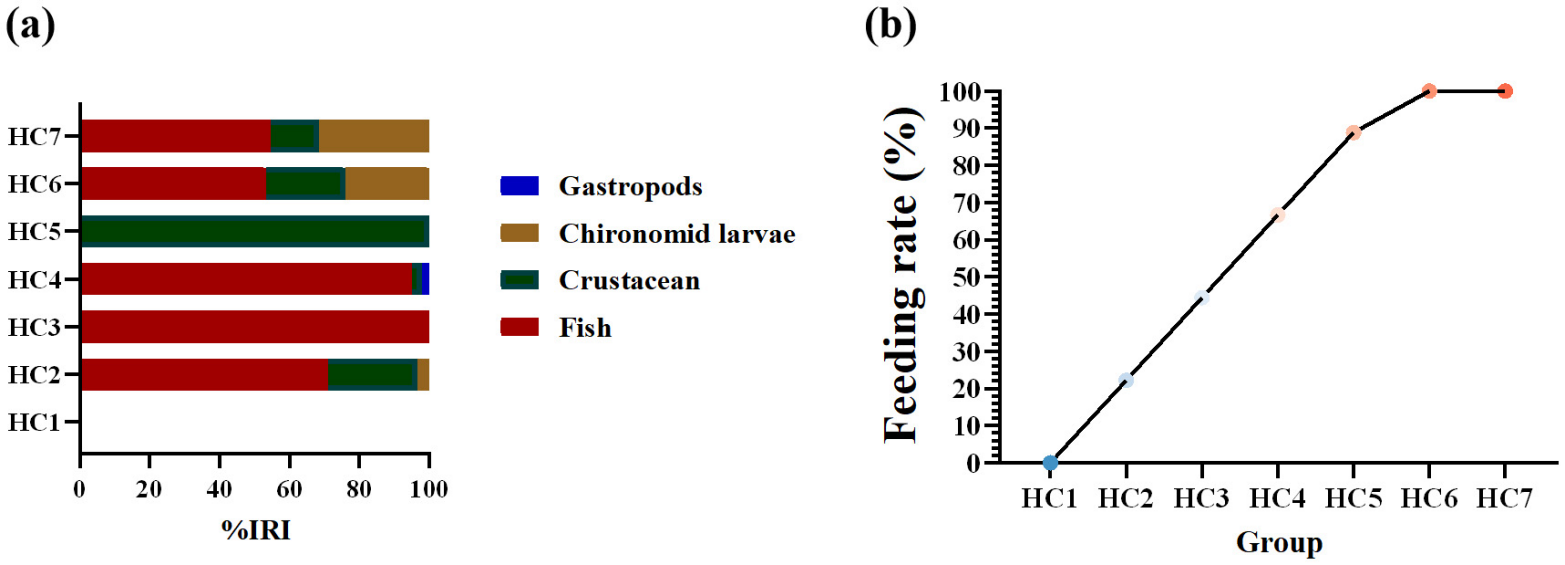
Relative importance index (IRI) percentage of prey species in the diet of *Huso dauricus* **(a)**. Feeding rate of *Huso dauricus* during environmental acclimation training **(b)**. HC1: day 2 of environmental acclimation training; HC2: day 5 of environmental acclimation training; HC3: day 10 of environmental acclimation training; HC4: day 15 of environmental acclimation training; HC5: day 20 of environmental acclimation training; HC6: day 25 of environmental acclimation training; HC7: day 30 of environmental acclimation training.

Relative importance index (IRI) percentage of prey species and feeding rate of *H. dauricus* during environmental acclimation are illustrated in Figure 2. In total, 72 fish were dissected (nine individuals per group). Prey items were classified into four categories: fish, chironomid larvae, crustaceans, and gastropods (Figure 2a). Feeding rate increased progressively throughout acclimation, first surpassing 50% in HC4 group and reaching its maximum in HC6 group (Figure 2b). In the HC1 group, no ingestion was observed in *H. dauricus*. The first feeding occurred on HC2 group with a feeding rate of 22.22%, and intestinal contents contained identifiable remains of three prey categories—fish, crustaceans, and chironomid larvae. In the HC3 group, only fish remains were found and the feeding rate was 44.44%. In the HC4 group, fish, crustaceans and, for the first time, gastropods were detected, raising the feeding rate to 66.67%. In the HC5 group, only crustacean remains were present, and the feeding rate increased to 88.89%. During the last two samplings-HC6 group and HC7 group-all stomachs examined contained three prey types (fish, chironomid larvae, and crustaceans), reaching a feeding rate of 100%. Therefore, HC1group, HC2-HC4 groups, and HC5-HC7 groups in this study are designated the fasting phase, the refeeding phase, and the stable-feeding phase, respectively.

### 3.2 Impact of environmental acclimation on digestive and immune enzyme activities

**Figure 3:**
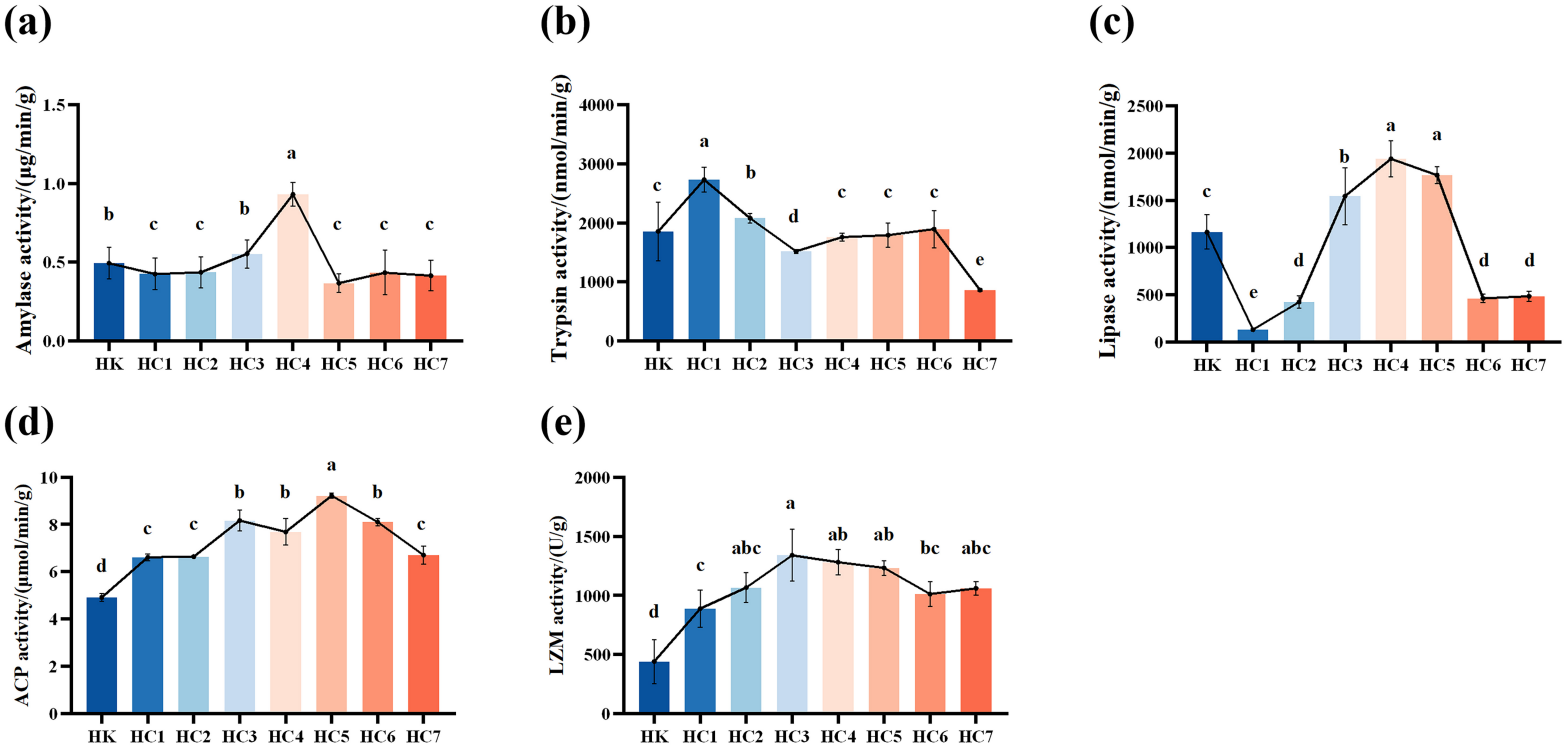
Effects of environmental acclimation training on digestive function and immunity of *Huso dauricus*. **(a)** Amylase activity. **(b)** Trypsin activity. **(c)** Lipase activity. **(d)** Acid phosphatase (ACP) activity. **(e)** lysozyme (LZM) activity. Bars represent mean ± SD; *n* = 9 individuals per group. Different small letters above the bars indicate significant differences (*P* < 0.05) in different groups. HK: cultured group; HC1: day 2 of environmental acclimation training; HC2: day 5 of environmental acclimation training; HC3: day 10 of environmental acclimation training; HC4: day 15 of environmental acclimation training; HC5: day 20 of environmental acclimation training; HC6: day 25 of environmental acclimation training; HC7: day 30 of environmental acclimation training.

Amylase activity showed a significant trend of first decreasing, then sharply increasing, and finally declining to a stable level with prolonged acclimation time (*P* < 0.05), reaching its peak in the HC4 group (Figure 3a). During the early acclimation period (groups HC1 and HC2), amylase activity did not differ significantly (*P* > 0.05). From HC3 group to HC4 group, activity increased markedly with acclimation time (*P* < 0.05), peaked in the HC4, and then declined to a stable level for the remainder of the experiment (*P* < 0.05). Trypsin activity rose significantly during the early acclimation phase and peaked in the HC1 group (*P* < 0.05), then declined markedly. No differences were detected among groups HC4, HC5 and HC6 relative to the control (*P* > 0.05), whereas activity in the HC7 was significantly lower than that of the control group (*P* < 0.05) (Figure 3b). Lipase activity was lowest in the HC1, and then increased markedly with acclimation time, peaked in the HC4, and fell to a steady level (*P* < 0.05; Figure 3c). The values stabilized in HC5 to HC7 groups and did not differ between groups HC6 and HC7 (*P* > 0.05).

ACP activity showed an initial increase followed by a decrease, reaching its maximum in HC5 group (Figure 3d). Notably, ACP activity was significantly higher than that of the control group during the entire environmental acclimation (*P* < 0.05). Similarly, LZM activity increased significantly and then decreased, peaking in HC3 group (Figure 3e). LZM activity was also significantly higher than that of the control group during the acclimation (*P* < 0.05).

### 3.3 Expression of immune-related genes

**Figure 4:**
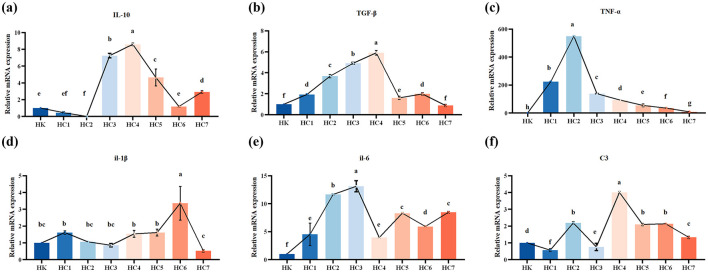
The mRNA expressions of immune-related genes in the intestine of the cultured (HK) and environmental acclimation training (HC) *Huso dauricus*. **(a)** *Interleukin-10* (*IL-10*); **(b)** *Transforming growth factor-beta* (*TGF*-β); **(c)** *Tumor necrosis factor-alpha* (*TNF*-α); **(d)** *Interleukin-1beta* (*IL-1*β); **(e)** *Interleukin-6* (*IL-6*); **(f)** *Complement 3* (*C3*). Different small letters above the bars indicate significant differences (*P* < 0.05) in different groups. HK: cultured group; HC1: day 2 of environmental acclimation training; HC2: day 5 of environmental acclimation training; HC3: day 10 of environmental acclimation training; HC4: day 15 of environmental acclimation training; HC5: day 20 of environmental acclimation training; HC6: day 25 of environmental acclimation training; HC7: day 30 of environmental acclimation training.

Compared with the cultured controls, the expression level of *Interleukin-10* (*IL-10*), *Transforming growth factor-beta* (*TGF-*β), *Tumor necrosis factor-*α*lpha* (*TNF-*α), *Interleukin-1beta* (*IL-1*β), *Interleukin-6* (*IL-6*), and *Complement 3* (*C3*) in gut of *H. dauricus* were significantly different throughout the acclimation period (Figure 4; *P* < 0.05). *IL-10* mRNA abundance showed a significant fluctuation during acclimation, first decreasing, then increasing, and finally decreasing again (*P* < 0.05). *TGF-*β mRNA levels in the environmentally acclimated group were significantly higher than those in the cultured group throughout the experiment (*P* < 0.05), and were shown to increase initially and then decrease with peaking in the HC4 group. *TNF-*α showed a similar trend, reaching its maximum in the HC2 group. In the early and mid stages of environmental acclimation, *IL-1*β mRNA expression levels did not differ significantly (*P* > 0.05), but increased markedly and then declined in the late stage (*P* < 0.05). Overall, the *IL-6* and *C3* mRNA levels rose significantly and subsequently decreased during the acclimation (*P* < 0.05).

### 3.4 Impact of environmental acclimation on intestinal microbiota

#### 3.4.1 Alpha-diversity analysis

Alpha-diversity indices of the intestinal microbiota for the HK and HC groups are presented in Table 2. Good's coverage exceeded 99.50% for all samples, indicating that sequencing depth was sufficient and the sampling of the intestinal microbiota was close to complete. Ace and Chao1 indices were highest in the HC7 group, followed by HC3-HC6, and lowest in HC2. Shannon and Simpson indices showed no significant differences between the HK and HC groups (*P* > 0.05).

**Table 2:** Alpha-diversity indices of intestinal microbiota in *Huso dauricus* from the control group and the field-acclimated group.

**Sample**	**Richness**		**Diversity**		**Coverage/%**
	**Ace**	**Chao 1**	**Shannon**	**Simpson**	**Coverage**
HK	362.05 ± 120.16^ab^	365.60 ± 119.42^a^	3.53 ± 0.33^a^	0.07 ± 0.01^a^	0.99945
HC1	312.04 ± 11.94^ab^	310.31 ± 215.11^a^	2.23 ± 0.77^a^	0.29 ± 0.10^a^	0.99889
HC2	144.97 ± 44.94^b^	138.54 ± 48.86^a^	1.33 ± 1.23^a^	0.51 ± 0.41^a^	0.99941
HC3	426.26 ± 265.76^ab^	431.89 ± 273.54^a^	2.39 ± 1.28^a^	0.34 ± 0.31^a^	0.99826
HC4	406.73 ± 36.28^a^	378.74 ± 70.58^a^	2.06 ± 1.84^a^	0.48 ± 0.41^a^	0.9985
HC5	557.08 ± 161.48^ab^	552.67 ± 150.01^a^	3.17 ± 1.56^a^	0.24 ± 0.35^a^	0.99772
HC6	427.72 ± 614.10^ab^	425.76 ± 617.65^a^	1.83 ± 2.63^a^	0.60 ± 0.51^a^	0.9982
HC7	955.71 ± 575.28^ab^	933.37 ± 555.21^a^	2.60 ± 1.29^a^	0.39 ± 0.32^a^	0.99535

Different lowercase letters represented significant differences (*P* < 0.05) among different group.

#### 3.4.2 Beta-diversity analysis

**Figure 5:**
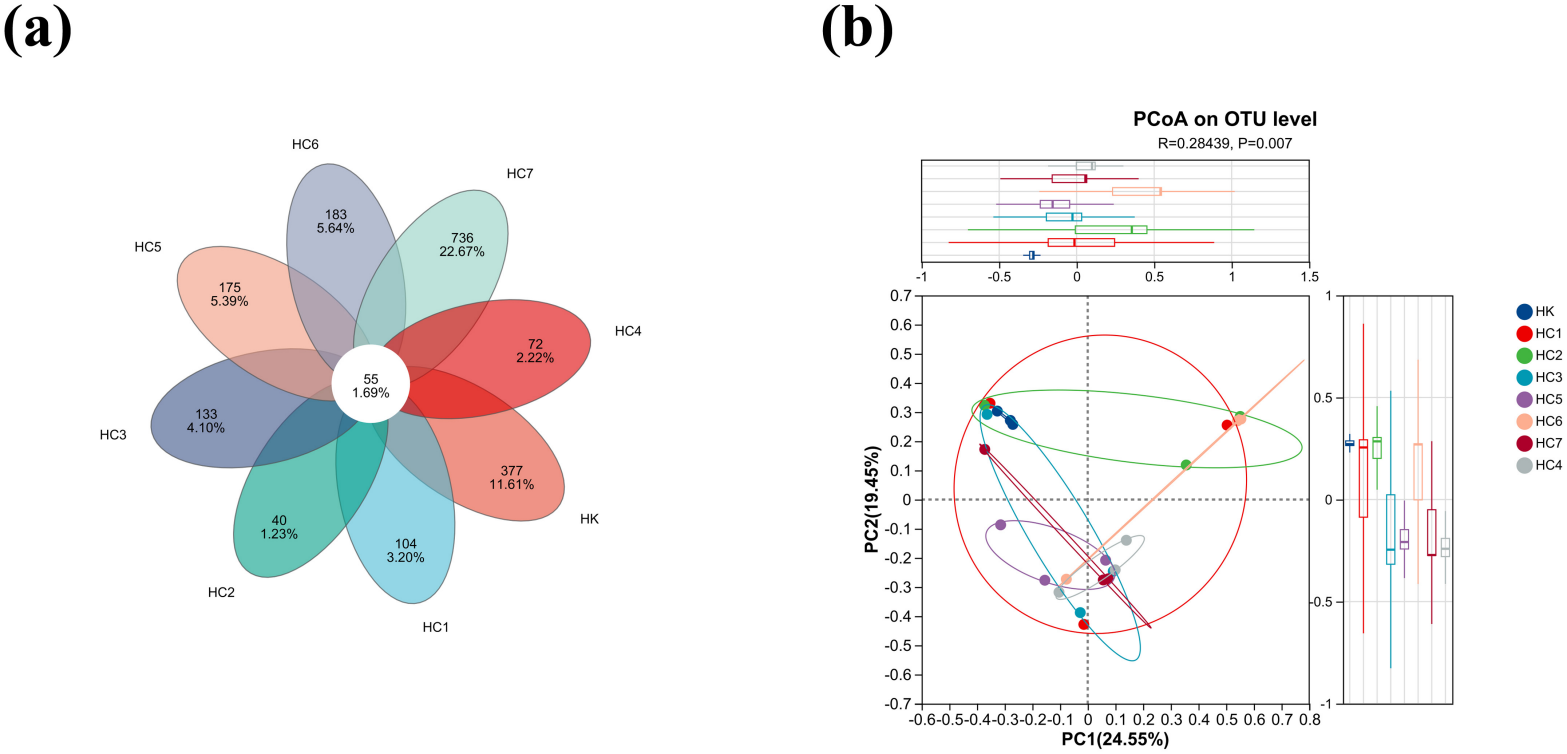
The intestinal microbe changes in *Huso dauricus* during environmental acclimation training (for *n* = 3 biological replicates). **(a)** Venn diagram for shared and specific OTUs among two groups. **(b)** Analysis of beta diversity of the intestinal microbiota.

As shown in Figure 5a, a total of 3,355 OTUs were detected across the eight groups. Among them, 432, 159, 95, 188, 127, 230, 238, and 791 unique OTUs were recorded in HK, HC1, HC2, HC3, HC4, HC5, HC6, and HC7, respectively, while 55 OTUs were shared by all groups (Figure 5a). The large difference in OTU counts between HK and HC7 indicates a pronounced divergence in microbial composition between the two groups. Meanwhile, Principal-coordinate analysis (PCoA) based on weighted UniFrac distances revealed a clear separation for microbiota between the HK and HC7 groups (*P* < 0.05; Figure 5b), indicating that environmental acclimation induced a substantial shift in community composition and increased microbial richness.

#### 3.4.3 Taxonomic composition of the intestinal microbiota

**Figure 6:**
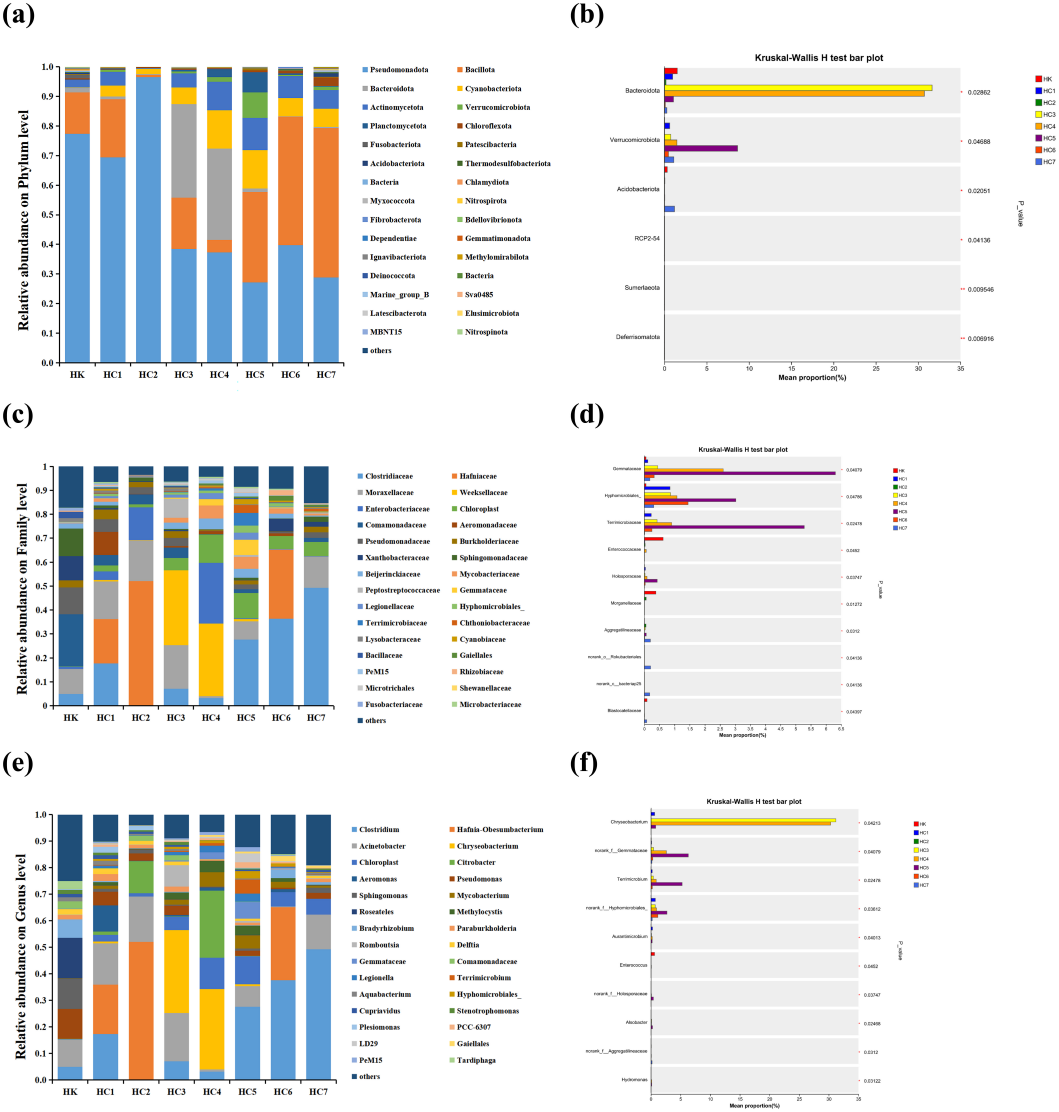
Differences in the relative abundance of the intestine bacteria flora of cultured (HK) and environmental acclimation training (HC) *Huso dauricus*. **(a)** Relative abundance of intestinal bacteria at the phylum level. **(b)** The significant bacterial community abundance at the phylum level. Values marked with asterisks are significantly different (*P < 0.05; **P < 0.01). **(c)** Relative abundance of intestinal bacteria at the family level. **(d)** The significant bacterial community abundance at the family level. Values marked with asterisks are significantly different (*P < 0.05). **(e)** Relative abundance of intestinal bacteria at the genus level. **(f)** The significant bacterial community abundance at the genus level. Values marked with asterisks are significantly different (*P < 0.05).

Comparative analysis of the eight intestinal communities identified a total of 44 phyla, 485 families and 1,003 genera. The mean and standard deviations for the dominant phyla, families and genera are shown in Supplementary Table S4. At the phylum level, Pseudomonadota dominated the communities of HK, HC1, HC2, HC3, and HC4 groups, accounting for 77.37%, 69.44%, 96.51%, 38.43%, and 37.23% of sequences, respectively (Figure 6a). In contrast, Bacillota became the most abundant taxon in groups HC5 (30.57%), HC6 (43.46%), and HC7 (50.55%), whereas Bacteroidota showed a marked expansion specifically in HC3 and HC4 groups.

At the family level (Figure 6c), Comamonadaceae dominated the HK group (21.80%), whereas Hafniaceae prevailed in HC1 (18.63%) and HC2 (52.03%). Weeksellaceae became the most abundant family in HC3 (31.24%) and HC4 (30.32%), and Clostridiaceae dominated HC5 (27.69%), HC6 (36.41%) and HC7 (49.24%). Notably, the relative abundance of Weeksellaceae increased markedly in HC3 and HC4 groups (*P* < 0.05).

At the genus level (Figure 6e), *Roseateles* dominated the HK group (15.25%), whereas *Hafnia-Obesumbacterium* prevailed in HC1 (18.63%) and HC2 (52.03%). *Chryseobacterium* became the most abundant genus in HC3 (31.21%) and HC4 (30.32%), and *Clostridium* dominated HC5 (27.69%), HC6 (37.65%) and HC7 (49.24%). Notably, the relative abundance of *Chryseobacterium* increased markedly in HC3 and HC4 groups (*P* < 0.05). After the fish were released into the natural environment, the intestinal microbiota shifted progressively with acclimation time. The transition occurred between HC3 and HC4, was completed by HC5, and remained stable in HC5-HC7. These results indicated that the gut-community shift began at days 10–15 (groups HC3-HC4) and was fully established by day 20 (HC5 group), after which it remained constant.

#### 3.4.4 Comparative analysis for differentially abundant gut microbiota

Significant differences were detected at three taxonomic levels (Figures 6b, d, f). At the phylum level, six taxa—Bacteroidota, Verrucomicrobiota, Acidobacteriota, RCP2-54, Sumerlaeota and Deferrisomatota—differed between groups (*P* < 0.05). Family-level analysis revealed 10 discriminatory lineages: Gemmataceae, Hyphomicrobiales, Terrimicrobiaceae, Enterococcaceae, Hiolosporaceae, Morganellaceae, Aggregatilineaceae, norank-o-Rokubacteriales, norank-c-bacteriap25 and Blastocatellaceae (*P* < 0.05). Among genera, ten taxa showed significant shifts: n*orank-f-Gemmataceae, Terrimicrobium, norank-f-Hyphomicrobiales, Aurantimicrobium, Enterococcus, norank-f-Holosporaceae, Alsobacter, norank-f-Aggregatilineaceae* and *Hydromonas* (*P* < 0.05).

#### 3.4.5 Functional prediction of gut microbiota

**Figure 7:**
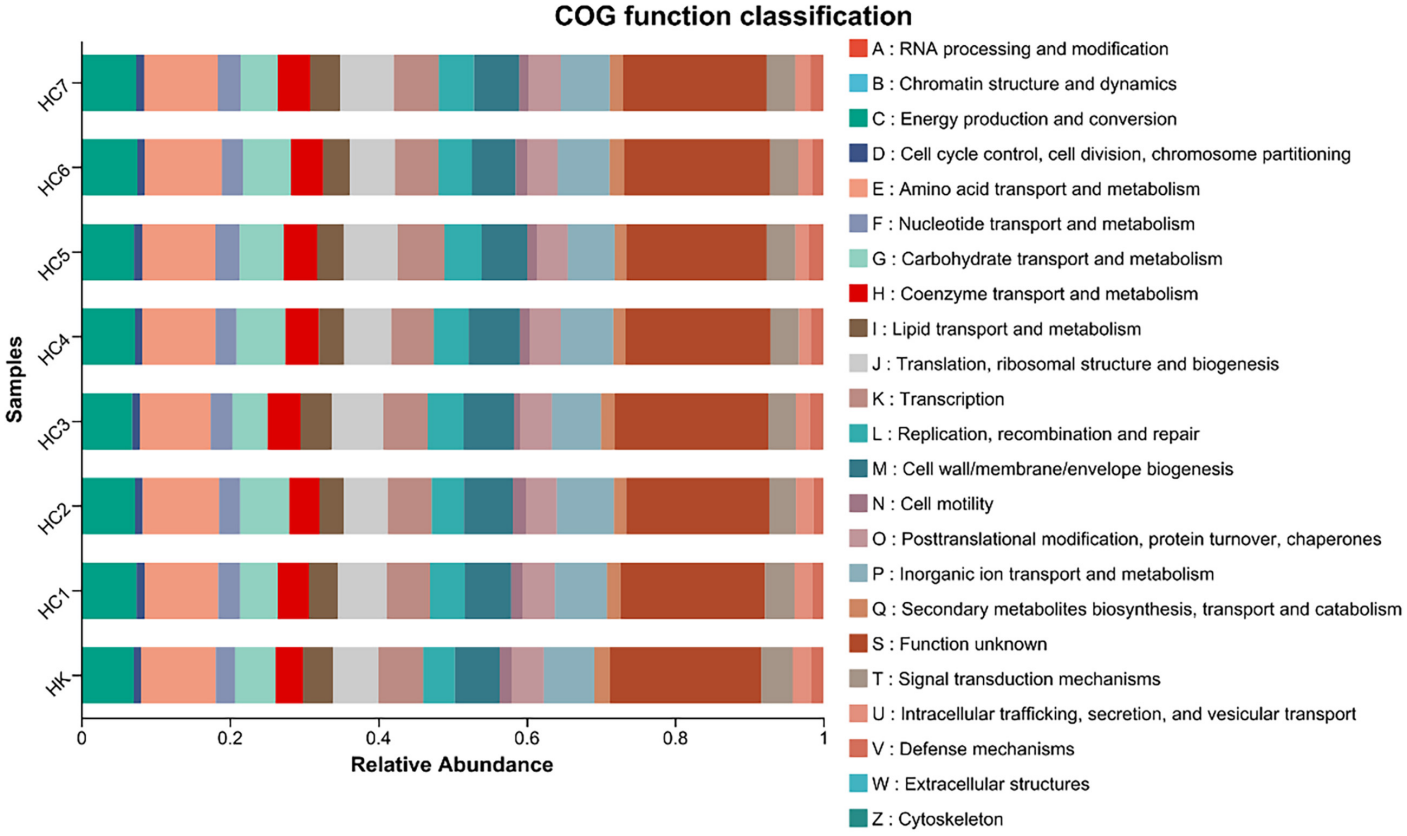
COG category distribution during environmental acclimation training (for *n* = 3 biological replicates).

Using PICRUSt2 high-precision functional inference, we predicted the shifts in intestinal microbial functions between the wild-conditioned and culture groups (Figure 7). COG-category abundance profiling revealed significant enrichment of microbial genes involved in energy production and conversion (C), amino-acid transport and metabolism (E), carbohydrate transport and metabolism (G), lipid transport and metabolism (I), secondary metabolites biosynthesis, transport and catabolism (Q), and defense mechanisms (V) in the environmental acclimation training groups and control group.

## 4 Discussion

### 4.1 Fourteen Days needed for natural prey capture in *H. dauricus*

Environmental acclimation training refers to a short-term, outdoor rearing phase in natural waters immediately prior to release, designed to allow captive-bred individuals to gradually adjust to ambient environmental conditions. The complexity of the natural environment directly modulates fish' foraging efficiency and anti-predator response capacity (Allen-Ankins et al., 2012; Jiang et al., 2025). A substantial body of field evidence indicates that hatchery-reared fish often fail to integrate into natural food webs, primarily because of ineffective anti-predator responses that elevate post-release mortality (Svasand and Kristiansen, 1990; Campton et al., 1991; Cámara-Ruiz et al., 2019). In the present study, *H. dauricus* did not feed during the early phase of environmental acclimation and minimal ingestion commenced on day 5 (HC2 group). Li et al. (2025) reported that farmed sturgeon did not prey within the first 7 d and commenced feeding between 14 and 30 d post-release, a pattern consistent with our observations, indicating that approximately 2 weeks are required for hatchery-reared *H. dauricus* to switch from culture to natural foraging mode. Likewise, studies on diet transition in sturgeons report that 5–8 d are required to accept novel diets and that normal feeding resumes approximately 10–15 d (Chen and Zhou, 1998). It is hypothesized that prolonged reliance on artificial pellets weakens natural prey-capture ability and reduces sensitivity to live prey, forcing fish into a temporary fasting state. In the initial phase after release, trout also fail to switch immediately to natural prey, and the presence of stones and detritus in their stomachs indicates that some individuals have difficulty discriminating food items in the wild (O'Grady, 1983). This further demonstrated that prolonged exposure to artificial diets under culture conditions attenuates innate foraging ability, so that newly released fish cannot capture live prey during the initial post-stocking. Therefore, an acclimation interval is required before successful feeding can occur. Targeted pre-release environmental acclimation is essential to enhance the survival of captive *H. dauricus*.

### 4.2 Digestive enzyme dynamics reflect dietary transition during acclimation

Fish mobilize endogenous energy reserves by modulating digestive enzyme activities, thereby sustaining routine physiological functions (Navarro-Guillén and Yúfera, 2025). Variations in digestive enzyme activities serve as an indicator for the relationship between fish growth and environmental conditions or dietary nutrient availability (Dutil et al., 1998). Modulating digestive enzyme activity is a stress response triggered by food scarcity (Pal and Maitra, 2018). When dietary energy is insufficient, fish undergo adaptive metabolic shifts that mobilize endogenous reserves (e.g., hepatic and muscle glycogen) to sustain vital functions while concurrently altering digestive enzyme activities (Saiz et al., 2021; Pérez-Jiménez et al., 2012). During starvation, digestive enzymes exhibit species- and duration-dependent changes. For example, protease, lipase and amylase activities all declined in juvenile hybrid grouper (*Epinephelus fuscoguttatus* ♀ × *Epinephelus lanceolatus* ♂) subjected to fasting (Zhang et al., 2022), whereas trypsin decreased continuously in *Megalobrama pellegrini*, amylase first rose then fell, and lipase decreased before increasing, all returning to control levels after refeeding (Li W. et al., 2021). Similarly, herring (*Clupea harengus*) larvae showed an initial rise followed by a decline in amylase activity under short-term starvation (Pedersen and Andersen, 1992). Collectively, these data indicated that fluctuations in digestive enzyme activity were contingent upon both species and the severity of food deprivation.

Previous studies have reported significant up-regulation of key metabolic enzymes during starvation periods in fish (Zhang et al., 2022). In the starvation phase of environmental acclimation, trypsin activity in the intestine of *H. dauricus* first increased and then declined, a trend comparable to that reported for trout (*Oncorhynchus mykiss*) (Furné et al., 2008). Additionally, *H. dauricus* subsisted almost exclusively on fish, crustacean and chironomid larvae tissues during the starvation phase of environmental acclimation, resulting in a protein-rich diet. Therefore, it was hypothesized that proteins constitute the primary energy source for *H. dauricus* in the early starvation period of environmental acclimation. In the present study, amylase activity declined during days 2–5 of environmental acclimation (HC1-HC2), increased between days 5 and 15 (HC2-HC4), and then decreased to a stable level for the remainder of the 30 d period (HC5-HC7). This pattern mirrors findings reported for sturgeon (*Acipenser naccarii*), in which amylase decreased during starvation and initially rose before declining to baseline after refeeding (Furné et al., 2008). This indicated that starch constituted the primary energy source sustaining normal metabolic activity of *H. dauricus* during refeeding. Additionally, during refeeding, lipase activity rose significantly, peaked on day 15 (HC4 group), and then declined to a stable level. These results suggested that both starch and lipids were mobilized to supply the energy required for *H. dauricus* to adapt to the natural environment during the refeeding phase.

Following the resumption of feeding, amylase, trypsin and lipase activities stabilized but remained significantly lower than the corresponding control values; similar results have been reported for orange-spotted grouper (*Epinephelus coioides*) (Wang et al., 2021). Previous studies have shown that starvation induces structural alterations in the liver of fish, leading to reduced protease secretion from these organs, and that enzyme activity recovers slowly, often failing to return to baseline levels during refeeding (Ehrlich et al., 1976). It was therefore hypothesized that starvation stress damages the digestive architecture of *H. dauricus*, impairs overall digestive function and consequently prevents digestive enzyme activities from fully recovering to their initial values after refeeding.

### 4.3 Immune response dynamics during environmental acclimation

Immune responses serve as a health indicator for fish in both wild and aquaculture settings, providing protection against pathogens and contributing to improved welfare (Ahi et al., 2025). ACP is a key non-specific immune enzyme that catalyzes dephosphorylation reactions, thereby facilitating signal transduction, metabolic regulation and environmental adaptation (Liang et al., 2014; Meng et al., 2024; Kong et al., 2012). Elevated ACP activity further indicates that defense against xenobiotics has been enhanced (Zang et al., 2012; Huo et al., 2018). LZM is a key component of innate immunity that lyses bacterial cell walls and prevents systemic infection (Canicatti and Roch, 1989). Its activity rapidly adjusts to environmental fluctuations, reflecting the immunological cost of altered habitat conditions (Langston et al., 2002; Li et al., 2019). In this study, both ACP and LZM activities were significantly higher in the environmentally acclimated group than in the control, most likely because the fish had not yet fully adapted to the new habitat and consequently up-regulated non-specific immune factors to counteract potential environmental hazards and pathogens. Previous studies have demonstrated that elevated LZM and ACP activities enhance immune competence in angtze sturgeon (*Acipenser dabryanus*) (Chen et al., 2019). Overall, environmental acclimation training markedly enhanced the immune competence of *H. dauricus*, thereby facilitating its adaptation to natural conditions. Inflammatory cytokines serve as key indicators of the intestinal immune barrier in aquatic vertebrates (Standen et al., 2016) and are commonly classified into pro- and anti-inflammatory subsets according to their functional effects (Baker et al., 2008). *TNF-*α and *IL-6* are key pro-inflammatory cytokines routinely used as biomarkers of inflammation (Ambroz et al., 2022), whereas anti-inflammatory mediators such as *IL-10* and *TGF-*β suppress the inflammatory response in fish (Zhang et al., 2021). In the present study, anti-inflammatory genes *IL-10* and *TGF-*β were significantly down-regulated, whereas the pro-inflammatory gene *IL-6* was up-regulated after 15 d of environmental acclimation (HC4 group) relative to the control group. Meanwhile, the expression of the pro-inflammatory gene *TNF-*α declined markedly after 10 d of training (HC3 group). These results further indicated that acclimation enhanced anti-inflammatory capacity and improved environmental adaptability, supporting the recommendation that Kaluga sturgeon should be released after approximately 15 d of conditioning, as also concluded by Li et al. (2025).

### 4.4 Gut microbiota succession during acclimation

Gut microbial community structure is increasingly recognized as an adaptive trait closely linked to the success of stocking programmes (West et al., 2019; Pan et al., 2025). The intestinal microbiota of fish performs critical functions, modulating host physiology, development and immune defense (Bates et al., 2006; Rawls et al., 2004; Kataoka, 2016). Owing to the direct continuity between ambient water and the fish intestinal milieu (Daly et al., 2019), fluctuations in natural aquatic conditions readily disrupt gut microbial balance (Robinson et al., 2018; Forehand et al., 2025). In this study, ACE, Chao 1 indexes and beta-diversity analyses revealed significant differences in richness, diversity and community structure between the gut microbiota of farmed and environmentally acclimated *H. dauricus*. Comparable divergence has been documented in Trinidadian guppies (Sullam et al., 2015). Diet-driven environmental factors have been shown to exert a strong influence on both alpha- and beta-diversity of gut microbiota (Round and Mazmanian, 2010; Liu et al., 2016). It was hypothesized that the diet shift experienced by *H. dauricus* necessitated a restructuring of the gut microbial community to cope with natural conditions, resulting in a significant increase in microbial richness. Moreover, the surrounding water contains a rich diversity of microorganisms, and these environmental microbes are introduced directly into the gut during feeding in *H. dauricus*, further enriching the intestinal community.

Numerous studies have identified Pseudomonadota and Bacillota as the dominant phyla in the intestinal microbiota of sturgeons (Geraylou et al., 2013; Xu et al., 2018), a result consistent with our study. At the phylum level, the gut microbiota of groups HK, HC1, HC2, HC3, and HC4 was dominated by Pseudomonadota, whereas Bacillota became the predominant phylum in groups HC5, HC6, and HC7. We therefore proposed that day 20 of environmental acclimation (HC5 group) represented the critical transition from hatchery to wild-type adaptation in *H. dauricus*, and that the dominance of Bacillota could serve as a biomarker indicating stabilization of the gut microbiota during the environmental acclimation process. Pseudomonadota, a dominant phylum of Gram-negative bacteria, plays a pivotal role in establishing the gut microbial community by consuming oxygen and lowering intestinal redox potential, thereby creating conditions conducive to the subsequent colonization of strict anaerobes (Shin et al., 2015). The elevated abundance of Pseudomonadota in the culture group (HK) likely reflects the high load of this phylum in the rearing water, where it has been implicated in organic-matter degradation and nitrogen fixation (Shen et al., 2020; Zhang et al., 2016). Groups HC3 and HC4 represented a turning point in the dominant microbiota, with a significant increase in the relative abundance of Bacteroidota compared with all other groups. Bacteroidota enhance digestion by degrading polysaccharides and proteins, thereby increasing the assimilative efficiency of nutrients (Spence et al., 2006). Their elevated abundance therefore implies an improved capacity for energy harvest, a response most likely necessitated by the low and irregular food intake of groups HC3 and HC4 (feeding rates 44.44% and 66.67%, respectively). It was also found that there was a increase in “energy production and conversion,” “carbohydrate transport and metabolism,” and “lipid transport and metabolism.” In the HC3 group, trypsin activity reached its nadir while the relative abundance of Bacteroidota increased significantly. This indicated that the reduced dietary protein supply lowered the demand for luminal proteolysis, whereas the enriched Bacteroidota efficiently hydrolyzed fish-muscle glycogen and residual chitin through their encoded glycanases and proteases, generating glucose and short-chain fatty acids (SCFA) (Ndeh et al., 2025). Under these conditions, the fish were required to digest and utilize ingested material more efficiently to maximize energy gain, representing a natural adaptation of *H. dauricus* to the fluctuating wild environment. Moreover, members of Bacteroidota can modulate T-cell proliferation and secrete polysaccharide A to suppress excessive immune responses (Zeng et al., 2019), thereby enhancing host immune function. Consistent with the microbial shift, HC3 and HC4 also showed the strongest immune response: AKP and LZM activities were significantly elevated, anti-inflammatory cytokines were up-regulated, and pro-inflammatory cytokines were markedly suppressed compared with the other groups. These results indicated that individuals in groups HC3 and HC4 had initiated a positive adaptation to the wild by modulating gut microbial composition to enhance food-use efficiency and strengthen immunity. Additionally, the decline in *TNF-*α after day 10 (HC3 group) therefore reflects a successful shift from an acute inflammatory state, triggered by the novel diet, water microbiota and handling stress, toward a homeostatic anti-inflammatory balance that is consistent with improved intestinal barrier function and overall environmental adaptation (Lee and Kang, 2017).

Accumulating evidence indicates that members of Bacillota play pivotal roles in the metabolism, digestion and absorption of proteins and other nutrients (Berry, 2016). For example, this phylum is associated with fatty-acid catabolism and produces elevated levels of butyrate, a metabolite recognized for its health-promoting properties (Kong et al., 2013). Post-mortem examination of stomach contents revealed undigested fish and crustacean remains rich in protein and lipid in groups HC5, HC6, and HC7, and it was therefore hypothesized that this high-protein diet drove Bacillota to dominance during this phase. Additionally, the abundance of Verrucomicrobiota increased significantly in group HC5, a shift that may reflect improved host metabolism, as previous studies have demonstrated that proliferation of this phylum is often associated with enhanced metabolic performance (Guo et al., 2018). Collectively, after 20 d of environmental acclimation (group HC5), the gut microbiota of *H. dauricus* stabilized and intestinal health was superior to that of both the control and earlier acclimation stages, identifying this time-point as optimal for pre-release conditioning.

## 5 Conclusion

In summary, this study demonstrated pronounced differences in gut health between farmed and environmentally acclimated *H. dauricus*, manifesting as a shift in energy-metabolism patterns and enhanced immune competence following acclimation. We therefore recommend a 20 day pre-release environmental acclimation that exposes *H. dauricus* to simulated natural conditions, thereby maintaining a healthy gut microbiota and maximizing post-stocking survival. These findings not only advance our understanding of the relationships among diet, health, and intestinal microbiota composition of *H. dauricus* during environmental acclimation, but also provide guidance for the potential success of future re-introduction efforts, providing a practical framework for the conservation of *H. dauricus* and other endangered critically fish.

## Data Availability

The datasets presented in this study can be found in online repositories. The names of the repository/repositories and accession number(s) can be found below: https://www.ncbi.nlm.nih.gov/bioproject/PRJNA1366084.

## References

[B1] Ahi E. P. Lindeza A. S. Miettinen A. Primmer C. R. (2025). Transcriptional responses to changing environments: insights from salmonids. *Rev. Fish Biol*. Fish. 35, 681–706. doi: 10.1007/s11160-025-09928-9

[B2] Allen-Ankins S. Stoffels R. J. Pridmore P. A. Vogel M. T. (2012). The effects of turbidity, prey density and environmental complexity on the feeding of juvenile Murray cod Maccullochella peelii. J. Fish Biol. 80, 195–206. doi: 10.1111/j.1095-8649.2011.03166.x 22220898

[B3] Ambroz A. Rossner P. Jr. Rossnerova A. Honkova K. Milcova A. Pastorkova A. . (2022). Oxidative stress and antioxidant response in populations of the czech republic exposed to various levels of environmental pollutants. Int. J. Environ. Res. Public Health 19:3609. doi: 10.3390/ijerph19063609 35329296 PMC8955578

[B4] Bacanu G. M. Oprea L. (2013). Differences in the gut microbiota between wild and domestic *Acipenser ruthenus* evaluated by denaturing gradient gel electrophoresis. *Rom. Biotechnol*. Lett. 18, 8069–8076.

[B5] Baker O. J. Camden J. M. Redman R. S. Jones J. E. Seye C. I. Erb L. (2008). Proinflammatory cytokines tumor necrosis factor-a and interferon-g alter tight junction structure and function in the rat parotid gland Par-C10 cell line. Am. J. Physiol. Cell. Ph. 295, C1191–201. doi: 10.1152/ajpcell.00144.2008 PMC2584989 18768927

[B6] Banerjee G. Ray A. K. (2017). Bacterial symbiosis in the fish gut and its role in health and metabolism. Symbiosis 72, 1–11. doi: 10.1007/s13199-016-0441-8

[B7] Bates J. M. Mittege E. Kuhlman J. Baden K. N. Cheesman S. E. (2006). Distinct signals from the microbiota promote different aspects of zebrafish gut differentiation. Dev. Biol. 297, 374–386. doi: 10.1016/j.ydbio.2006.05.006 16781702

[B8] Beamish R. J. Thompson B. L. McFarlane G. A. (1992). Spiny dogfish predation on chinook and coho salmon and the potential effects on hatchery-produced salmon. Trans. Am. Fish. Soc. 37, 805–811. doi: 10.1577/1548-8659(1992)121%3C0444:SDPOCA%3E2.3.CO;2

[B9] Berry D. (2016). The emerging view of firmicutes as key fibre degraders in the human gut. Environ. Microbiol. 18, 2081–2083. doi: 10.1111/1462-2920.13225 26842002

[B10] Bolnick D. I. Snowberg L. K. Hirsch P. E. Lauber C. L. Knight R. Caporaso J. G. . (2014). Individuals' diet diversity influences gut microbial diversity in two freshwater fish (threespine stickleback and Eurasian perch). Ecol. Lett. 17, 979–987. doi: 10.1111/ele.12301 24847735 PMC4084827

[B11] Brown C. Day R. L. (2002). The future of stock enhancements: lessons for hatchery practice from conservation biology. Fish Fish. 3, 79–94. doi: 10.1046/j.1467-2979.2002.00077.x

[B12] Cámara-Ruiz M. Santo C. E. Gessner J. Wuertz S. (2019). How to improve foraging efficiency for restocking measures of juvenile Baltic sturgeon (*Acipenser oxyrinchus*). Aquaculture 502, 12–17. doi: 10.1016/j.aquaculture.2018.12.021

[B13] Campton D. E. Allendorf F. W. Behnke R. J. Utter F. M. Chilcote M. W. Leider S. A. (1991). Reproductive success of hatchery and wild steelhead. Trans. Am. Fish. Soc. 120, 816–827. doi: 10.1577/1548-8659(1991)120[816:RSOHAW]2.0.CO;2

[B14] Canicatti C. Roch P. (1989). Studies on Holothuria polii (*Echinodermata*) antibacterial proteins. I. Evidence for and activity of a coelomocyte lysozyme. Experientia 45, 756–759. doi: 10.1007/BF01974579

[B15] Chebanov M. Rosenthal H. Gessner J. van Anrooy R. Doukakis P. Pourkazemi M. . (2011). Sturgeon hatchery practices and management for release guidelines. *FAO Fish. Aquac. Tech*. Pap. 570, 90–93.

[B16] Chen H. Wang B. Yu N. Qi J. Tang N. Wang S. . (2019). Transcriptome analysis and the effects of polyunsaturated fatty acids on the immune responses of the critically endangered angtze sturgeon (*Acipenser dabryanus*). *Fish Shellfish Immunol*. 94, 199–210. doi: 10.1016/j.fsi.2019.09.012 31499199

[B17] Chen H. T. Zhou C. H. (1998). Trial on weaning Amur sturgeon (*Acipenser schrenckii*) larvae to artificial formulated diets. Heilongjiang Fish. 1, 17–18.

[B18] Chester R. E. Johnson L. A. Butler M. J. Sesnie S. E. Wolfe D. H. (2025). Conditioning effect on survival of foster parents of reintroduced masked bobwhite broods. Endanger. Species Res. 57, 33–44. doi: 10.3354/esr01404

[B19] Chong R. Grueber C. E. Fox S. Wise P. Barrs V. R. Hogg C. J. (2019). Looking like the locals-gut microbiome changes post-release in an endangered species. Anim. Microb. 1, 1–10. doi: 10.1186/s42523-019-0012-4 PMC7807427 33499935

[B20] Coleman F. Travis J. Thistle A. B. (1998). Marine stock enhancement: a new persective. B. Mar. Sci. 62:303.

[B21] Cowx I. G. (2025). Fish Stocking in Inland Waters in Europe and Central Asia: Issues and solutions. EIFAAC Occasional Paper, No. 54. Rome: Food and Agriculture Organization (FAO).

[B22] Daly K. Kelly J. Moran A. W. Bristow R. Young I. S. Cossins A. R. . (2019). Host selectively contributes to shaping intestinal microbiota of carnivorous and omnivorous fish. J. Gen. Appl. Microbiol. 65, 129–136. doi: 10.2323/jgam.2018.07.003 30416165

[B23] De Vos W. M. Tilg H. Van Hul M. Cani P. D. (2022). Gut microbiome and health: mechanistic insights. Gut 71, 1020–1032. doi: 10.1136/gutjnl-2021-326789 35105664 PMC8995832

[B24] Dehler C. E. Secombes C. J. Martin S. A. M. (2017). Environmental and physiological factors shape the gut microbiotaof Atlantic salmon parr (*Salmo salar* L.). Aquaculture 467, 149–157. doi: 10.1016/j.aquaculture.2016.07.017 28111483 PMC5142738

[B25] Dhanasiri A. K. Brunvold L. Brinchmann M. F. Korsnes K. Bergh Ø. Kiron V. (2011). Changes in the intestinal microbiota of wild Atlantic cod *Gadus morhua* L. upon captive rearing. Microb. Ecol. 61, 20–30. doi: 10.1007/s00248-010-9673-y 20424834

[B26] Dutil J. Lambert Y. Guderley H. E. Blier P. U. Pelletier D. Desroches M. (1998). Nucleic acids and enzymes in Atlantic cod (*Gadus morhua*) differing in condition and growth rate trajectories. Can. J. Fish. Aquat. Sci. 55, 788–795. doi: 10.1139/f97-294

[B27] Ehrlich K. F. Blaxter J. H. S. Pemberton R. (1976). Morphological and histological changes during the growth and starvation of herring and plaice larvae. Mar. Biol. 35, 105–118. doi: 10.1007/BF00390932

[B28] Feng Q. Chen W. D. Wang Y. D. (2018). Gut microbiota: an integral moderator in health and disease. *Front*. Microbiol. 9:151. doi: 10.3389/fmicb.2018.00151 PMC5826318 29515527

[B29] Forehand C. R. Smith S. N. Nielsen F. Bauer B. Watters J. L. Moody R. W. . (2025). Comparative assessment of Texas horned lizard (*Phrynosoma cornutum*) gut microbiome diversity and composition throughout transition from captivity to wild. Front. Microbiomes 4:1601442. doi: 10.3389/frmbi.2025.1601442 PMC12993635 41852402

[B30] Furné M. García-Gallego M. Hidalgo M. C. Morales A. E. Domezain A. Domezain J. . (2008). Effect of starvation and refeeding on digestive enzyme activities in sturgeon (*Acipenser naccarii*) and trout (*Oncorhynchus mykiss*). Comp. Biochem. Physiol. A Mol. Integr. Physiol. 149, 420–425. doi: 10.1016/j.cbpa.2008.02.002 18328757

[B31] Geraylou Z. Souffreau C. Rurangwa E. De Meester L. Courtin C. M. Delcour J. A. . (2013). Effects of dietary arabinoxylan-oligosaccharides (AXOS) and endogenous probiotics on the growth performance, non-specific immunity and gut microbiota of juvenile Siberian sturgeon (*Acipenser baerii*). *Fish Shellfish Immunol*. 35, 766–775. doi: 10.1016/j.fsi.2013.06.014 23811408

[B32] Gessner J. Arndt G. M. Fredrich F. Ludwig A. Kirschbaum F. Bartel R. . (2011). “Biology and Conservation of the European Sturgeon Acipenser sturio L. 1758,” in The Reunion of the European and Atlantic Sturgeons, Remediation of Atlantic sturgeon Acipenser Oxyrinchus in the Oder River: Background and First Results, eds. P. Williot, E. Rochard, N. Desse-Berset, F. Kirschbaum, and J. Gessner (Berlin/Heidelberg: Springer), 539–559.

[B33] Guo J. Han X. Zhan J. You Y. Huang W. (2018). Vanillin alleviates high fat diet-induced obesity and improves the gut microbiota composition. Front. Microbiol. 9:2733. doi: 10.3389/fmicb.2018.02733 30483238 PMC6243071

[B34] Humphries P. King A. McCasker N. Kopf R. K. Stoffels R. Zampatti B. . (2020). Riverscape recruitment: a conceptual synthesis of drivers of fish recruitment in rivers. Can. J. Fish. Aquat. Sci. 77, 213–225. doi: 10.1139/cjfas-2018-0138

[B35] Huo D. Sun L. Ru X. Zhang L. Lin C. Liu S. . (2018). Impact of hypoxia stress on the physiological responses of sea cucumber *Apostichopus japonicus*: respiration, digestion, immunity and oxidative damage. *PeerJ* 6:e4651. doi: 10.7717/peerj.4651 PMC5926553 29719735

[B36] Hutchison M. Butcher A. Norris A. (2023). Conditioning to predators improves survival of stocked Murray cod (*Maccullochella peelii*) fingerlings. Mar. Freshw. Res. 74, 1039–1049. doi: 10.1071/MF22242

[B37] Jiang J. Zhou S. Song J. Xia C. Yang X. Yang K. . (2025). Diet-microbiome coevolution: the core mechanism for semi-aquatic adaptation and cross-habitat niche coexistence of the web-footed shrew (Nectogale elegans). Front. Microbiol. 16:1711143. 41321819 10.3389/fmicb.2025.1711143 PMC12659910

[B38] Kataoka K. (2016). The intestinal microbiota and its role in human health and disease. J. Med. Invest. 63, 27–37. doi: 10.2152/jmi.63.27 27040049

[B39] Kong L. C. Tap J. Aron-Wisnewsky J. Pelloux V. Basdevant A. Bouillot J. L. . (2013). Gut microbiota after gastric bypass in human obesity: increased richness and associations of bacterial genera with adipose tissue genes. Am. J. Clin. Nutr. 98, 16–24. doi: 10.3945/ajcn.113.058743 23719559

[B40] Kong X. Wang S. Jiang H. Nie G. Li X. (2012). Responses of acid/alkaline phosphatase, lysozyme, and catalase activities and lipid peroxidation to mercury exposure during the embryonic development of goldfish Carassius auratus. Aquat. Toxicol. 120, 11–125. doi: 10.1016/j.aquatox.2012.05.005 22683699

[B41] Koshelev V. N. Vilkova O.Y. Kotsyuk V. (2022). Modern data on the distribution, abundance and qualitative structure of the populations of the Kaluga *Huso dauricus* and the Amur sturgeon *Acipenser schrenckii* (*Acipenserida*e) in the Amur River and the Amur Estuary. J. Ichthyol. 62, 1394–1403. doi: 10.1134/S0032945222070037

[B42] Langston A. L. Hoare R. Stefansson M. Fitzgerald R. Wergeland H. Mulcahy M. (2002). The effect of temperature on non-specific defence parameters of three strains of juvenile Atlantic halibut (*Hippoglossus hippoglossus* L.). *Fish Shellfish Immunol*. 12, 61–76. doi: 10.1006/fsim.2001.0354 11866131

[B43] Le Vay L. Carvalho G. R. Quinitio E. T. Lebata J. H. Ut V. N. Fushimi H. (2007). Quality of hatchery-reared juveniles for marine fisheries stock enhancement. Aquaculture 268, 169–180. doi: 10.1016/j.aquaculture.2007.04.041

[B44] Lee S. I. Kang K. S. (2017). Function of capric acid in cyclophosphamide-induced intestinal inflammation, oxidative stress, and barrier function in pigs. Sci. Rep. 7:16530. doi: 10.1038/s41598-017-16561-5 29184078 PMC5705592

[B45] Li J. Wang C. Pan W. Du H. Zhang H. Wu J. . (2021). Migration and distribution of adult hatchery reared Yangtze sturgeons (*Acipenser dabryanus*) after releasing in the upper Yangtze River and its implications for stock enhancement. J. Appl. Ichthyol. 37, 3–11. doi: 10.1111/jai.14117

[B46] Li W. Ma X. Wang W. (2021). Ontogeny of digestive enzymes and the effect of short-term starvation on enzyme activities in *Megalobrama pellegrini* larvae. J. Shanghai Ocean Univ. 30, 612–620.

[B47] Li X. Ji L. Wu L. Gao X. Li X. Li J. . (2019). Effect of flow velocity on the growth, stress and immune responses of turbot (*Scophthalmus maximus*) in recirculating aquaculture systems. Fish Shellfish Immunol. 86, 1169–1176. doi: 10.1016/j.fsi.2018.12.066 30599254

[B48] Li Y. Wang R. Zhai C. Cao D. Sun Z. Zhang Y. . (2025). Dynamic impacts of stock enhancement on Kaluga sturgeon (*Huso dauricus*): novel conservation strategy insights from the gut microbe composition and gene expression mode. Int. J. Mol. Sci. 26:1480. doi: 10.3390/ijms26041480 40003945 PMC11855664

[B49] Liang S. Luo X. You W. Luo L. Ke C. (2014). The role of hybridization in improving the immune response and thermal tolerance of abalone. Fish Shellfish Immunol. 39, 69–77. doi: 10.1016/j.fsi.2014.04.014 24794582

[B50] Liu H. Guo X. Gooneratne R. Lai R. Zeng C. Zhan F. Wang W. (2016). The gut microbiome and degradation enzyme activity of wild freshwater fishes influenced by their trophic levels. Sci. Rep. 6, 24340. doi: 10.1038/srep24340 PMC4829839 27072196

[B51] Liu H. B. Zhang Y. Lv T. Y. Wang D. Zhao J. W. (2010). Analysis of papain hydrolyzed fragments of immunoglobulin in serum of Chinese sturgeon *Acipenser sinesis* gray and Huso sturgeon Georgi. Chin. J. Fish. 23, 7–10.

[B52] Lv S. Zhao W. Shi Z. Wang S. Wei J. (2018). Comparative study of the intestinal microbial community of wild and cultured kaluga sturgeon, *Huso dauricus. Aquac*. Res. 49, 2938–2944. doi: 10.1111/are.13712

[B53] McNeil W. (1991). Expansion of cultured Pacific salmon into marine ecosystems. Aquaculture 98, 173–183. doi: 10.1016/0044-8486(91)90382-H

[B54] Meng X. Luo L. Zhao Z. Wang S. Zhang R. Guo K. (2024). Ginger polysaccharide alleviates the effects of acute exposure to carbonate in crucian carp (*Carassius auratus*) by regulating immunity, intestinal microbiota, and intestinal metabolism. Ecotoxicol. Environ. Saf. 273:116127. doi: 10.1016/j.ecoenv.2024.116127 38394756

[B55] Navarro-Guillén C. Yúfera M. (2025). Understanding rhythms in the digestive functionality of fish gut. J. Exp. Biol. 228:jeb249942. doi: 10.1242/jeb.249942 40698423

[B56] Ndeh D. A. Nakjang S. Kwiatkowski K. J. Sawyers C. Koropatkin N. M. Hirt R. P. . (2025). A *Bacteroides thetaiotaomicron* genetic locus encodes activities consistent with mucin O-glycoprotein processing and N-acetylgalactosamine metabolism. Nat. Commun. 16:3485. doi: 10.1038/s41467-025-58660-2 40216766 PMC11992087

[B57] Nickleson T. E. (1986). Influences of upwelling, ocean temperature and smolt abundance in marine survival of coho salmon (*Oncorhynchus kistuch*) in the Oregon production area. Can. J. Fish. Aquat. Sci. 43, 527–535. doi: 10.1139/f86-063

[B58] O'Grady M. F. (1983). Observations on the dietary habits of wild and stocked brown trout, *Salmo trutta* L., in Irish lakes. *J. Fish Biol. 22*, 593–601. doi: 10.1111/j.1095-8649.1983.tb04219.x

[B59] Pal P. K. Maitra S. K. (2018). Response of gastrointestinal melatonin, antioxidants, and digestive enzymes to altered feeding conditions in carp (*Catla catla*). Fish Physiol. Biochem. 44, 1061–1073. doi: 10.1007/s10695-018-0494-0 29572613

[B60] Pan H. Liu H. Liu F. Xie J. Zhou Y. Zheng Q. . (2025). Gut microbiota: a new frontier in understanding and protecting endangered plateau schizothorax fish. Front. Microbiol. 16:1592312. doi: 10.3389/fmicb.2025.1592312 40584040 PMC12202599

[B61] Pearcy W. G. (1992). Ocean Ecology of North Pacific Salmonids. Seattle: University of Washington Press. 179 p.

[B62] Pedersen B. H. Andersen N. G. (1992). Induction of trypsinogen and chymotrypsinogen secretion and growth in herring (*Clupea harengus*) larvae. Fish Physiol. Biochem. 10, 1–11.24214190

[B63] Pérez-Jiménez A. Cárdenas S. García-Alonso J. Abellán E. (2012). Effects of fasting and refeeding on digestive enzyme activities and antioxidant status in common dentex (*Dentex dentex*). *Aquaculture* 356–357, 1–8.

[B64] Pinos A. Alonso-Alonso P. Correa-Carmona Y. Holzmann K. L. Yon F. Brehm G. . (2025). Host identity, more than elevation, shapes bee microbiomes along a tropical elevation gradient. Front. Microbiol. 16:1671348. 41048498 10.3389/fmicb.2025.1671348 PMC12488565

[B65] Rawls J. F. Samuel B. S. Gordon J. I. (2004). Gnotobiotic zebrafish reveal evolutionarily conserved responses to the gut microbiota. Proc. Natl. Acad. Sci. U.S.A. 101, 4596–4601. doi: 10.1073/pnas.0400706101 15070763 PMC384792

[B66] Ringø E. Zhou Z. Vecino J. L. G. Wadsworth S. Romero J. Krogdahl Å. . (2016). Effect of dietary components on the gut microbiota of aquatic animals. A never-ending story? Aquacult. Nutr. 22, 219–282. doi: 10.1111/anu.12346

[B67] Robinson C. D. Klein H. S. Murphy K. D. Parthasarathy R. Guillemin K. Bohannan B. J. M. (2018). Experimental bacterial adaptation to the zebrafish gut reveals a primary role for immigration. PLoS Biol. 16:e2006893. doi: 10.1371/journal.pbio.2006893 30532251 PMC6301714

[B68] Rochard E. Castelnaud G. Lepage M. (1990). Sturgeons (Pisces: *Acipenseridae*); threats and prospects. J. Fish Biol. 37, 123–132. doi: 10.1111/j.1095-8649.1990.tb05028.x

[B69] Round J. L. Mazmanian S. K. (2010). The gut microbiota shapes intestinalimmune responses during health and disease. *Nat. Rev*. Drug Discov. 5, 9–16. doi: 10.1038/nri2515 PMC4095778 19343057

[B70] Ruzauskas M. Armalyte J. Lastauskiene E. ŠiugŽdiniene R. Klimiene I. Mockeliunas R. . (2021). Microbial and antimicrobial resistance profiles of microbiota in common carps (*Cyprinus carpio*) from aquacultured and wild fish populations. Animals 11:929. doi: 10.3390/ani11040929 33805887 PMC8064328

[B71] Saiz F. Velasco C. Míguez J. M. Soengas J. L. (2021). Metabolic effects of REV-ERBα activation in rainbow trout (*Oncorhynchus mykiss*): role of hepatic glucose and lipid metabolism. Comp. Biochem. Physiol. 247:109043.

[B72] Salvanes A. G. V. (2001). “Ocean ranching,” in Encyclopedia of Ocean Sciences, Vol. 4, eds. J. Steele, K. K. Turkian, and S.A. Thorpe (New York: Academic Press), 1973–1982.

[B73] Shen X. Xu M. Li M. Zhao Y. Shao X. (2020). Response of sediment bacterial communities to the drainage of wastewater from aquaculture ponds in different seasons. Sci. Total Environ. 717:137180. doi: 10.1016/j.scitotenv.2020.137180 32065893

[B74] Shin N. R. Whon T. W. Bae J. W. (2015). Proteobacteria: microbial signature of dysbiosis in gut microbiota. Trends Biotechnol. 33, 496–503. doi: 10.1016/j.tibtech.2015.06.011 26210164

[B75] Song H. L. Zhu B. Y. Dong T. Wang W. Hu M. Yan X. Y. . (2023). Whole-genome resequencing reveals selection signatures for caviar yield in Russian sturgeon (*Acipenser gueldenstaedtii*). *Aquaculture* 568:739312. doi: 10.1016/j.aquaculture.2023.739312

[B76] Spence C. Wells W. G. Smith C. J. (2006). Characterization of the primary starch utilization operon in the obligate anaerobe *Bacteroides fragilis*: regulation by carbon source and oxygen. J. Bacteriol. 188, 4663–4672. doi: 10.1128/JB.00125-06 16788175 PMC1482989

[B77] Standen B. T. Peggs D. T. Rawling M. D. Foey A. Davies S. J. Santos G. A. . (2016). Dietary administration of a commercial mixed-species probiotic improves growth performance and modulates the intestinal immunity of tilapia, *Oreochromis niloticus*. Fish Shellfish Immunol. 49, 427–435. doi: 10.1016/j.fsi.2015.11.037 26672904

[B78] Steven A. M. (2010). Rebuilding depleted fish stocks: the good, the bad, and, mostly, the ugly. ICES J. Mar. Sci. 67, 1830–1840. doi: 10.1093/icesjms/fsq125

[B79] Su G. Logez M. Xu J. Tao S. Villéger S. Brosse S. (2021). Human impacts on global freshwater fish biodiversity. Science 371, 835–838. doi: 10.1126/science.abd3369 33602854

[B80] Sullam K. E. Essinger S. D. Lozupone C. A. O'Connor M. P. Rosen G. L. Knight R. . (2012). Environmental and ecological factors that shape the gut bacterial communities of fish: a meta-analysis. Mol. Ecol. 21, 3363–3378. doi: 10.1111/j.1365-294X.2012.05552.x 22486918 PMC3882143

[B81] Sullam K. E. Rubin B. E. Dalton C. M. Kilham S. S. Flecker A. S. Russell J. A. (2015). Divergence across diet, time and populations rules out parallel evolution in the gut microbiomes of Trinidadian guppies. ISME J. 9, 1508–1522. doi: 10.1038/ismej.2014.231 25575311 PMC4478690

[B82] Svasand T. Kristiansen T. S. (1990). Enhancement studies of coastal cod in western Norway. Part IV. Mortality of reared cod after release. J. Cons. 47, 30–39. doi: 10.1093/icesjms/47.1.30

[B83] Tomiyama T. Watanabe M. Kawata G. Ebe K. (2011). Post-release feeding and growth of hatchery-reared Japanese flounder Paralichthys olivaceus: relevance to stocking effectiveness. J. Fish Biol. 78, 1423–1436. doi: 10.1111/j.1095-8649.2011.02949.x 21539551

[B84] Wang J. Li S. W. Sun Z. P. Lu C. Y. Zhao R. Liu T. Q. . (2024). Comparative study of immune responses and intestinal microbiota in the gut-liver axis between wild and farmed pike perch (*Sander Lucioperca*). *Front. Immunol*. 15:1473686. doi: 10.3389/fimmu.2024.1473686 PMC11494242 39439785

[B85] Wang Q. Li M. Chen R. (2021). Effects of feed deprivation and re-feeding on digestive enzymes and growth performance in juvenile orange-spotted grouper, *Epinephelus coioides. Fish Physiol*. Biochem. 47, 171–183.

[B86] Wei Q. W. Ke F. E. Zhang J. M. Zhuang P. Luo J. D. Zhou R. Q. . (1997). Biology, fisheries, and conservation of sturgeons and paddlefish in China. Environ. Biol. Fish. 48, 241–255. doi: 10.1023/A:1007395612241

[B87] Wei Q. Ruban G. (2010). “*Huso dauricus*,” in IUCN 2012 IUCN Red List of Threatened Species. Version 2012.1. Gland: International Union for Conservation of Nature (IUCN). Available online at: www.iucnredlist.org

[B88] Wen C. Yan W. Mai C. Duan Z. Zheng J. Sun C. . (2021). Joint contributions of the gut microbiota and host genetics to feed efficiency in chickens. Microbiome 9:126. doi: 10.1186/s40168-021-01040-x 34074340 PMC8171024

[B89] West A. G. Waite D. W. Deines P. Bourne D. G. Digby A. McKenzie V. J. . (2019). The microbiome in threatened species conservation. Biol. Conserv. 229, 85–98. doi: 10.1016/j.biocon.2018.11.016

[B90] Xu G. L. Xing W. Li T. L. Ma Z. H. Liu C. X. Jiang N. . (2018). Effects of dietary raffinose on growth, non-specific immunity, intestinal morphology and microbiome of juvenile hybrid sturgeon (*Acipenser baeri* Brandt ♀ × A. schrenckii Brandt ♂). Fish Shellfish Immunol. 72, 237–246. doi: 10.1016/j.fsi.2017.11.001 29104091

[B91] Xu X. M. Yuan Y. B. Wang Z. L. Zheng T. Cai H. T. Yi M. L. . (2023). Environmental DNA metabarcoding reveals the impacts of anthropogenic pollution on multitrophic aquatic communities across an urban river of western China. Environ. Res. 216:114512. doi: 10.1016/j.envres.2022.114512 36208790

[B92] Ye Y. Valbo-Jørgensen J. (2012). Effects of IUU fishing and stock enhancement on and restoration strategies for the stellate sturgeon fishery in the Caspian Sea. *Fish*. Res. 131, 21–29. doi: 10.1016/j.fishres.2012.06.022

[B93] Zang Y. Tian X. Dong S. Dong Y. (2012). Growth, metabolism and immune responses to evisceration and the regeneration of viscera in sea cucumber, Apostichopus japonicus. Aquaculture 358, 50–60. doi: 10.1016/j.aquaculture.2012.06.007

[B94] Zeng Q. Li D. He Y. Li Y. Yang Z. Zhao X. . (2019). Discrepant gut microbiota markers for the classification of obesity related metabolic abnormalities. Sci. Rep. 9:13424. doi: 10.1038/s41598-019-49462-w 31530820 PMC6748942

[B95] Zhang C. N. Jiang D. X. Wang J. H. Qi Q. (2021). The effects of TPT and dietary quercetin on growth, hepatic oxidative damage and apoptosis in zebrafish. Ecotoxicol. Environ. Saf. 224:112697. doi: 10.1016/j.ecoenv.2021.112697 34450426

[B96] Zhang H. Sun Z. Liu B. Xuan Y. Jiang M. Pan Y. . (2016). Dynamic changes of microbial communities in *Litopenaeus vannamei* cultures and the effects of environmental factors. Aquaculture 455, 97–108. doi: 10.1016/j.aquaculture.2016.01.011

[B97] Zhang K. Wang X. Liu H. (2022). Starvation-induced changes in digestive enzyme activities and energy metabolism of juvenile hybrid grouper (*Epinephelus fuscoguttatus* ♀ × *Epinephelus lanceolatus* ♂). *Aquaculture* 560:738494.

[B98] Zhuang P. Kynard B. Zhang L. Zhang T. Zhang Z. Li D. (2022). Overview of biology and aquaculture of Amur sturgeon (*Acipenser schrenckii*) in China. J. Appl. Ichthyol. 18, 659–664. doi: 10.1046/j.1439-0426.2002.00365.x

