# Supplementary material for: Pre-release environmental acclimation enhances wild adaptability of endangered Kaluga sturgeon (Huso dauricus): insights from digestive, immune, and gut-microbiome perspectives
Source: Front Microbiol. 2025 Dec 4;16:1720688. doi: 10.3389/fmicb.2025.1720688 (PMC12711814; doi:10.3389/fmicb.2025.1720688)
Supplement: Supplementary file 1 [file Supplementary_file_1.docx]

Table S1 The nutrient content of feed for Huso dauricus

| Nutrient content indicators (g/100g) | |
| --- | --- |
| Crude protein | 44.10 |
| Crude ether extract | 10.30 |
| Crude ash | 18.60 |
| Crude fiber | 5.80 |
| Ca | 4.90 |
| Total Phosphorus | 1.60 |
| Lysine | 2.30 |
| Water | 12.40 |

Table S2 Water quality parameters of wild training and farmed environments

| Group | Water temperature/℃ | Dissolved oxygen (DO)/(mg/L) | pH value |
| --- | --- | --- | --- |
| HC1 | 17.42±0.41 | 8.25±0.01 | 7.56±0.03 |
| HC2 | 14.78±0.18 | 8.11±0.28 | 8.09±0.43 |
| HC3 | 8.19±0.03 | 8.20±0.01 | 8.38±0.03 |
| HC4 | 6.57±0.06 | 8.01±0.00 | 8.92±0.02 |
| HC5 | 5.58±0.07 | 7.99±0.01 | 8.73±0.07 |
| HC6 | 2.30±0.02 | 8.01±0.00 | 9.06±0.03 |
| HC7 | 3.26±0.03 | 8.16±0.01 | 9.83±0.07 |
| HK | 13.13±0.12 | 7.70 ±0.10 | 8.51±0.17 |

**Table S3** The size of target fish for experiment.

| Sample | Body length/cm | Body weight/g |
| --- | --- | --- |
| HK | 16.14±0.71^a^ | 28.51±5.07^a^ |
| HC1 | 17.09±1.69^a^ | 27.34±7.51^a^ |
| HC2 | 15.85±1.08^a^ | 27.92±6.42^a^ |
| HC3 | 15.99±1.76^a^ | 27.77±7.47^a^ |
| HC4 | 15.60±1.28^a^ | 26.78±4.84^a^ |
| HC5 | 16.56±1.56^a^ | 28.51±6.68^a^ |
| HC6 | 16.27±1.05^a^ | 26.81±4.39^a^ |
| HC7 | 16.70±1.12^a^ | 31.85±5.70^a^ |

Table S4 **The mean and standard deviations for the dominant phyla, families and genera**

|  |  | HK | HC1 | HC2 | HC3 | HC4 | HC5 | HC6 | HC7 |
| --- | --- | --- | --- | --- | --- | --- | --- | --- | --- |
| phyla | Pseudomonadota | 77.3716065±9.0450 | 69.4408322±47.9100 | 96.5122253±5.3820 | 38.4321469±47.4400 | 37.2304901±41.7800 | 27.1741399±20.1100 | 39.6826728±31.9800 | 28.7859815±33.4600 |
|  | Bacillota | 14.0113022±5.7420 | 19.560436±33.3400 | 0.8035127±1.1160 | 17.3248888±23.6700 | 4.3177768±3.4440 | 30.5679976±47.1600 | 43.4612±3.4090 | 50.5450401±38.1100 |
|  | Bacteroidota | 1.5307679±1.0210 | 0.9736872±0.9651 | 0.1838206±0.1484 | 31.646037±44.2200 | 30.743791±52.0800 | 1.0788422±1.2140 | 0.1067604±0.9824 | 0.2913837±0.2366 |
|  | Cyanobacteriota | 0.1460932±0.1284 | 3.6467113±6.0560 | 1.7916486±3.0180 | 5.6655268±6.7540 | 12.9958741±18.5200 | 13.0103228±9.0790 | 6.2450834±10.7400 | 6.1447447±9.2400 |
|  | Actinomycetota | 2.4787683±1.6100 | 4.6685611±7.2280 | 0.3058325±0.4734 | 4.6509014±4.6820 | 9.6533898±13.3700 | 10.8791279±7.0470 | 7.3488096±12.7000 | 6.3895712±5.7550 |
| families | Clostridiaceae | 5.00409382074±8.3330 | 17.6580134534±30.3500 | 0.0594005362102±0.0947 | 7.12806434523±8.0200 | 3.18113952704±3.2810 | 27.6918878133±46.6100 | 36.41300481±1.1380 | 49.2422418083±38.7800 |
|  | Hafniaceae | 0.00240812984636±0.0041 | 18.6260816517±32.1800 | 52.0260399107±49.7300 | 0±000 | 0.00160541989757±0.0028 | 0±0.0000 | 28.7389482±54.6600 | 0±0.0000 |
|  | Moraxellaceae | 10.3108092922±3.7200 | 15.6046814044±25.8400 | 17.0230698839±29.2300 | 18.2078697683±31.2600 | 0.813145178121±0.9827 | 7.66106375122±8.6930 | 0.192650387709±0.1626 | 13.1588241905±22.4900 |
|  | Weeksellaceae | 0.113182102779±0.0995 | 0.644576088876±0.9424 | 0.0369246576442±0.0558 | 31.2390630769±44.5100 | 30.3191574756±52.4100 | 0.862110484997±1.3130 | 0.000802709948787±0.0014 | 0.03371381784910.0563 |
|  | Enterobacteriaceae | 0.856491515356±0.5499 | 3.62744625857±4.8750 | 13.7656729118±23.3500 | 0.0778628650323±0.0507 | 25.360818122±43.9100 | 0.342757148132±0.4515 | 0.0666249257493±0.0779 | 0.097930613752±0.1675 |
| genera | Clostridium | 5.00329111078797±8.3310 | 17.367432451944±29.8400 | 0.05940053621015±0.0947 | 7.09274510747757±7.9620 | 3.18113952704467±3.2810 | 27.6918878132603±46.6100 | 37.6473341327±1.1370 | 49.2414390983967±38.7800 |
|  | Hafnia-Obesumbacterium | 0.00240812984636±0.0042 | 18.6260816516515±32.1800 | 52.0260399107333±49.7300 | 0±0.0000 | 0.00160541989757333±0.0028 | 0±0.0000 | 27.5038162±54.6600 | 0±0.0000 |
|  | Acinetobacter | 10.2546195957667±3.6950 | 15.5814028159213±25.8200 | 16.9893560660903±29.1800 | 18.1910128594137±31.2500 | 0.797893689093933±0.9805 | 7.653839361701±8.6820 | 0.188636837965027±0.1583 | 13.1467835412407±22.4700 |
|  | Chryseobacterium | 0.0337138178490667±0.0379 | 0.637351699336267±0.9409 | 0.02729213825875±0.0392 | 31.210968228722±44.5400 | 30.3175520557418±52.4100 | 0.80993433832756±1.3300 | 0.000802709948786667±0.0014 | 0.0144487790781533±0.2230 |
|  | Chloroplast | 0.120406492318±0.1053 | 2.516495689446±4.1400 | 1.27149255887727±2.1230 | 5.067507906701±6.3040 | 11.8223121257427±17.1700 | 10.5259355584433±7.1420 | 5.44558429257663±9.3630 | 5.875836825123±9.0140 |

**Appendix 1**

1. α-Amylase

Definition: Endo-hydrolase that randomly cleaves α-1,4-glycosidic bonds in starch and glycogen, producing maltose, maltotriose and dextrins.

Principle: α-Amylase hydrolyses soluble starch to release reducing sugars, which reduce 3,5-dinitrosalicylic acid to generate the brown-red product 3-amino-5-nitrosalicylic acid; absorbance is read at 540 nm and is directly proportional to the amount of reducing sugar formed.

Unit: 1 U = the amount of enzyme that releases 1 µg of reducing sugar (calculated as maltose) per minute, per g of tissue (µg/min/g).

1. Lipase

Definition: One unit (U) of lipase activity is defined as the amount of enzyme that catalyses the release of 1 nmol of p-nitrophenol (p-NP) from the substrate per minute, per g of tissue, under the assay conditions.

Principle: Lipase hydrolyses p-NPP to yield p-nitrophenol, which exhibits a yellow colour with maximum absorbance at 405 nm. The rate of increase in A₄₀₅ nm is directly proportional to lipase activity.

Unit: nmol/min/g

1. Trypsin

Definition: One unit (U) of trypsin activity is defined as the amount of enzyme that release 1 nmol of p-nitroaniline per minute, per g of tissue, under the assay conditions.

Principle: Trypsin cleaves Nα-benzoyl-DL-arginine-p-nitroanilide (BAPNA) to release p-nitroaniline, which shows a yellow colour with maximum absorbance at 405 nm. The rate of increase in 405 nm is directly proportional to trypsin activity.

Unit: nmol/min/g

1. Acid phosphatase (ACP)

Definition: One unit (U) of acid phosphatase activity is defined as the amount of enzyme that hydrolyses 1 µmol of p-nitrophenyl phosphate (PNPP) to yield p-nitrophenol (PNP) per g of tissue, per minute, under the assay conditions.

Principle: Acid phosphatase hydrolyses PNPP to yield PNP, which exhibits a yellow colour with maximum absorbance at 405 nm. The rate of increase in 405 nm is directly proportional to acid phosphatase activity.

Unit: µmol/min/g

1. Lysozyme (LZM)

Principle: The kit employs the turbidimetric method using Micrococcus lysodeikticus as substrate. Lysozyme lyses the bacterial cell wall, reducing turbidity. The rate of decrease in absorbance at 530 nm is directly proportional to lysozyme activity.

Unit: U/g
